# Synthesis, structure, and reactivity of crystalline molecular complexes of the {[C_5_H_3_(SiMe_3_)_2_]_3_Th}^1–^ anion containing thorium in the formal +2 oxidation state†
†Electronic supplementary information (ESI) available: Experimental and computational details; crystallographic data collection, structure solution, and refinement; and crystallographic data and complete bond distances and angles for compounds **1–4**. CCDC 1018011–1018014. For ESI and crystallographic data in CIF or other electronic format see DOI: 10.1039/c4sc03033h



**DOI:** 10.1039/c4sc03033h

**Published:** 2014-11-03

**Authors:** Ryan R. Langeslay, Megan E. Fieser, Joseph W. Ziller, Filipp Furche, William J. Evans

**Affiliations:** a Department of Chemistry , University of California , Irvine , California 92697-2025 , USA . Email: wevans@uci.edu ; Email: filipp.furche@uci.edu ; Fax: +1-949-824-2210 ; Tel: +1-949-824-5174

## Abstract

Reduction of the Th^3+^ complex Cp′′3Th, **1** [Cp′′ = C_5_H_3_(SiMe_3_)_2_], with potassium graphite in THF in the presence of 2.2.2-cryptand generates [K(2.2.2-cryptand)][Cp′′3Th], **2**, a complex containing thorium in the formal +2 oxidation state. Reaction of **1** with KC_8_ in the presence of 18-crown-6 generates the analogous Th^2+^ compound, [K(18-crown-6)(THF)_2_][Cp′′3Th], **3**. Complexes **2** and **3** form dark green solutions in THF with *ε* = 23 000 M^–1^ cm^–1^, but crystallize as dichroic dark blue/red crystals. X-ray crystallography revealed that the anions in **2** and **3** have trigonal planar coordination geometries, with 2.521 and 2.525 Å Th–(Cp′′ ring centroid) distances, respectively, equivalent to the 2.520 Å distance measured in **1**. Density functional theory analysis of (Cp′′3Th)^1–^ is consistent with a 6d^2^ ground state, the first example of this transition metal electron configuration. Complex **3** reacts as a two-electron reductant with cyclooctatetraene to make Cp′′2Th(C_8_H_8_), **4**, and [K(18-crown-6)]Cp′′.

## Abstract 2

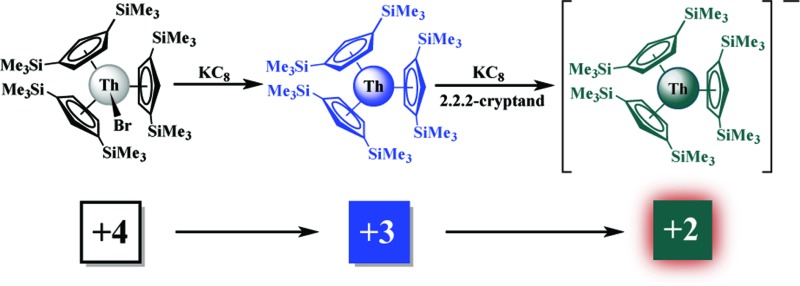
Structural, spectroscopic, and DFT analysis of the first molecular complexes of Th^2+^ indicate they have a 6d^2^ electron configuration of the type expected for the transactinide ions Rf^2+^ and Db^3+^.

## 

One of the fundamental characteristics of any metal is the extent to which it loses electrons to form charged species in different formal oxidation states. This ionization can occur in the gas phase to form short-lived species in a wide range of oxidation states, but the number of oxidation states available in solution in molecular metal complexes for productive chemistry is smaller. Chemists have tested the limits of oxidation states of all the elements for over 100 years and the boundaries of oxidation states accessible in solution are well established.

Nevertheless, it was recently discovered that the +2 oxidation state is accessible in soluble molecular complexes for all the elements in the lanthanide series except promethium, eqn (1).^1^ Previously, it was thought that only the traditional six Ln^2+^ ions of Eu, Yb, Sm, Tm, Dy, and Nd were obtainable in solution on the basis of calculated reduction potentials^2^ and solid state chemistry.^3^
1
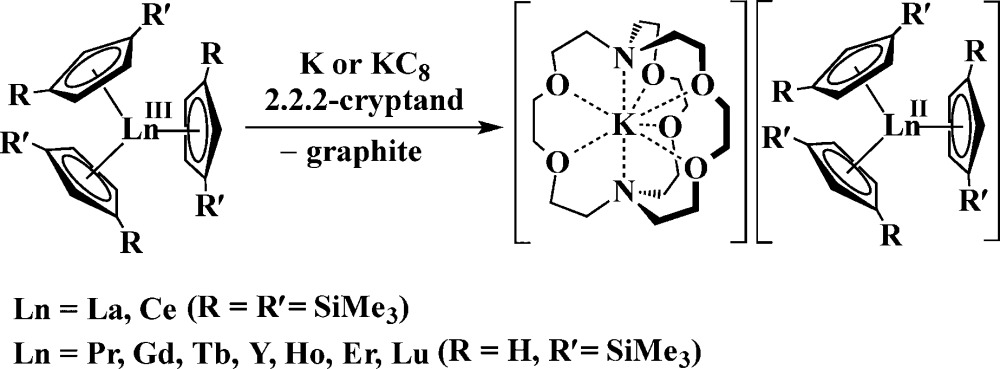



Extension of this reductive chemistry to uranium was not initially tried since it is well known that the redox chemistry of uranium, which includes multiple oxidation states, +3, +4, +5, and +6, is quite different from that of the rare earths. Although it was likely that uranium would be different, an analogous synthesis was eventually attempted and the first fully characterizable U^2+^ complex, [K(2.2.2-cryptand)][Cp′3U] (Cp′ = C_5_H_4_SiMe_3_), was isolated according to eqn (2).^4^
2
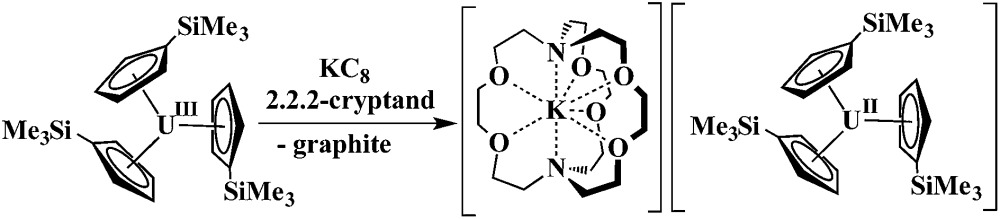



Synthesis of a Th^2+^ complex *via*eqn (1) or (2) seemed even more unlikely for several reasons. Complexes of Th^3+^ are already difficult to obtain. The Th^4+^/Th^3+^ redox potential is estimated to be –3.0 and –3.8 V *vs.* NHE^5^ and a Th^3+^/Th^2+^ redox potential of –4.9 V *vs.* NHE is in the literature.^6^ Reduction to metallic thorium would be predicted to be favored before formation of a Th^2+^ species.^6^ Many studies have been reported to find oxidation states lower than +4 for thorium,^7^ but only five Th^3+^ complexes have ever been structurally characterized.^7k–o^ An analog of eqn (2) was not possible since Cp′3Th has not yet been synthesized. Despite these issues, thorium reduction chemistry was examined using Cp′′3Th [Cp′′ = C_5_H_3_(SiMe_3_)_2_-1,3],^7*k*^ prepared by Lappert *et al.* in 1986, and the results are described here.

Addition of potassium graphite to a dark blue solution of Cp′′3Th, **1**, and 2.2.2-cryptand in THF immediately forms a green solution from which dichroic dark blue/red crystals of [K(2.2.2-cryptand)][Cp′′3Th], **2**, can be isolated and crystallographically characterized, Fig. 1, eqn (3). The analogous reaction with 18-crown-6 instead of 2.2.2-cryptand as the potassium chelator provides [K(18-crown-6)(THF)_2_][Cp′′3Th], **3**, which was also crystallographically characterized [see (ESI†)], eqn (3). Elemental analysis was consistent with the structures determined crystallographically. The ^1^H and ^13^C NMR spectra of **2** and **3** gave resonances in the diamagnetic region with a Me_3_Si ^1^H NMR resonance shifted about 0.4 ppm from that of KCp′′. A resonance was observed in the ^29^Si NMR spectrum of **3** at –6 ppm in the region close to the –8 and –15.5 ppm signals of diamagnetic Cp′′3ThBr and KCp′′, respectively. Evans method measurements^8^ on both **2** and **3** and SQUID analysis^9^ at low temperature suggest the [Cp′′3Th]^1–^ anion is diamagnetic. No EPR spectra were observed for **2** and **3**. Decomposed samples showed the EPR spectrum of Cp′′3Th.^7*m*^
3
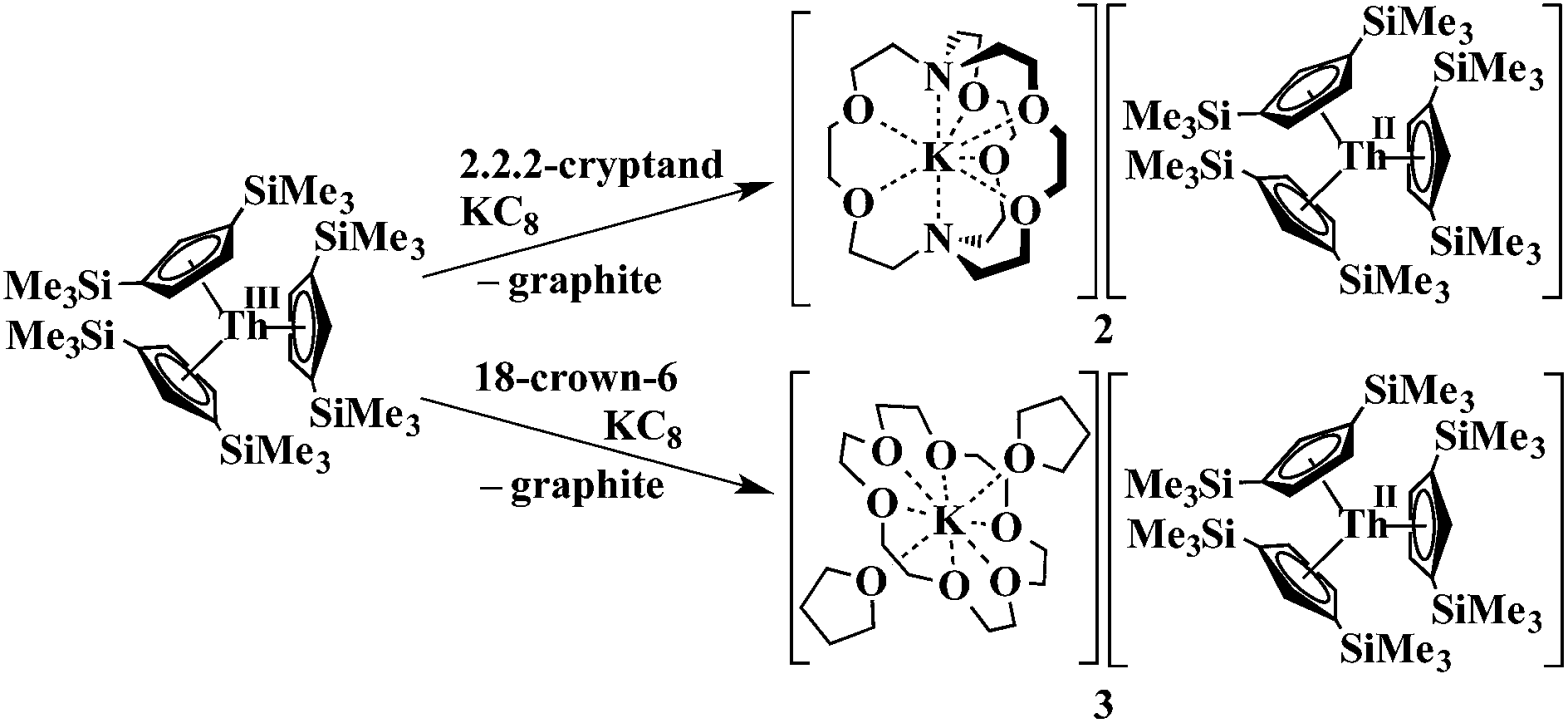



**Fig. 1:**
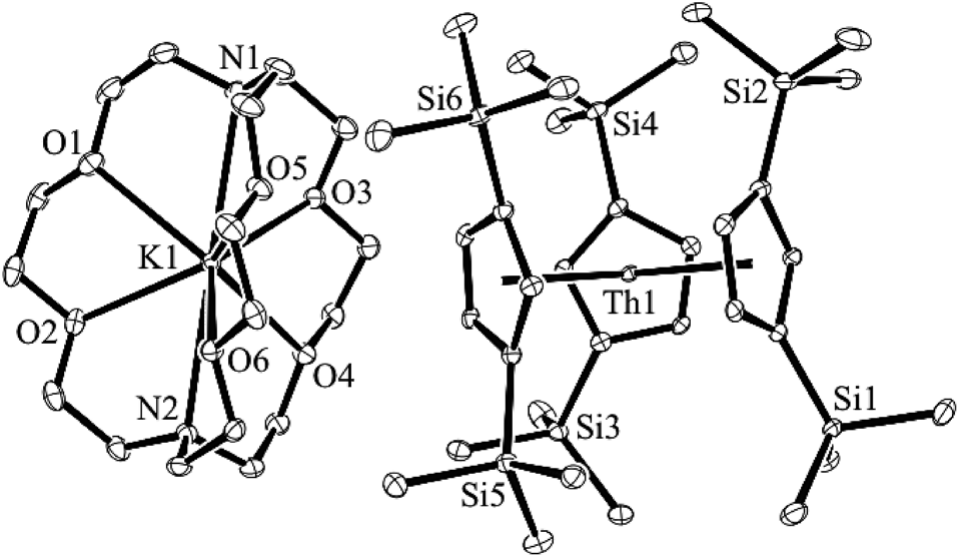
Molecular structure of [K(2.2.2-cryptand)][Cp′′3Th], **2**. Thermal ellipsoids are drawn at the 50% probability level and hydrogen atoms are omitted for clarity.

The structures of the anions in **2** and **3** are very similar to the structure of Cp′′3Th. All three structures have a trigonal planar arrangement of the three Cp′′ rings around thorium with a sum of (ring centroid)–Th–(ring centroid) angles of 360°. The structure of **2**, however, is not isomorphous with the lanthanum complex of the same formula, [K(2.2.2-cryptand)][Cp′′3La].^1*a*^ The average Th–(Cp′′ ring centroid) distances of 2.521 Å in **2** and 2.525 Å in **3** are equivalent to the 2.520 Å distance in Cp′′3Th. The negligible differences in the Th–(ring centroid) distances between the Th^3+^ precursor and the formally Th^2+^ complexes **2** and **3** are similar to the small differences between the Cp′3Ln and Cp′′3Ln Ln^3+^ complexes and the (Cp′3Ln)^1–^ and (Cp′′3Ln)^1–^ complexes, respectively, of all the new Ln^2+^ ions that have 4f^*n*^5d^1^ ground states^1^ instead of the 4f^*n*+1^ configurations expected by reduction of a 4f^*n*^ Ln^3+^ ion. Similarly, the 2.521 Å U–(ring centroid) distance in the U^2+^ complex, [K(2.2.2-cryptand)][Cp′3U], which appears to have a 5f^3^6d^1^ ground state, is only slightly larger than the 2.508 Å value in the U^3+^ analog, Cp′3U.^4^ These small changes in M–(ring centroid) distances match the small changes in radial size commonly seen in transition metal complexes,^10^ but contrast with the 0.10–0.20 Å differences generally seen for complexes of 4f^*n*+1^ Ln^2+^ complexes compared to their 4f^*n*^ Ln^3+^ counterparts.^11^

The UV-Vis spectra of **2** and **3** in THF, Fig. 2, contain absorptions at 650 nm with extinction coefficients of 23 000 M^–1^ cm^–1^, that are significantly larger than those of Cp′′3Th, 5000 M^–1^ cm^–1^. This is similar to the larger intensities observed for the +2 complexes, [K(2.2.2-cryptand)][Cp′3Ln]^1c,d^ and [K(2.2.2-cryptand)][Cp′3U],^4^ compared to their +3 analogs, Cp′3Ln and Cp′3U, respectively. However, the absorptions of the Th^2+^ complexes are even more intense and the solutions look like ink.

**Fig. 2:**
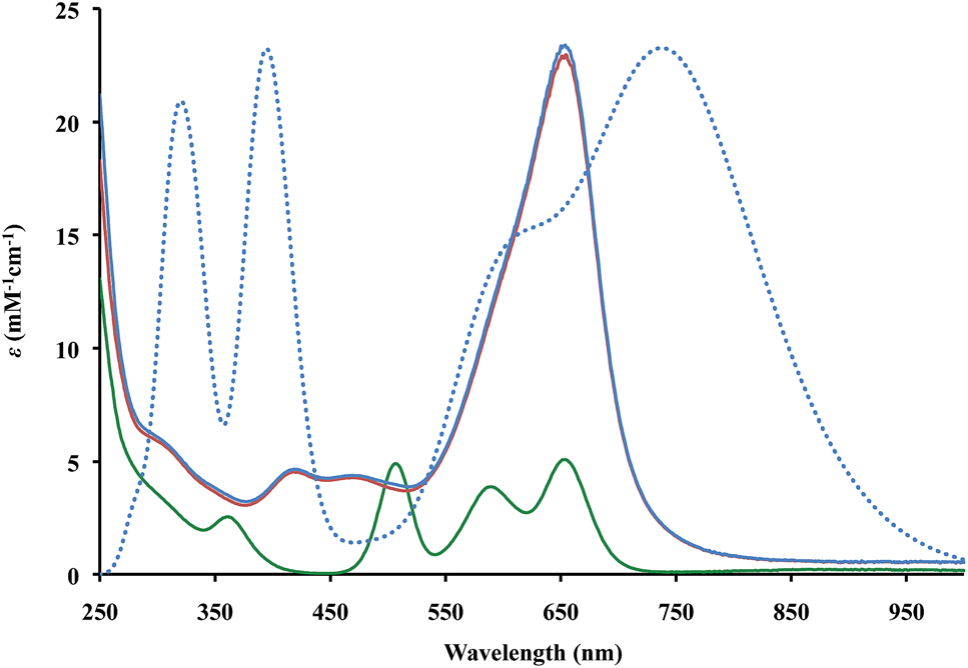
Experimental (solid lines) UV-Vis spectra in THF at 298 K for Cp′′3Th, **1** (green), [K(2.2.2-cryptand)][Cp′′3Th], **2** (red), and [K(18-crown-6)(THF)_2_][Cp′′3Th], **3** (blue) and calculated (dotted) UV-Vis spectra of (Cp′′3Th)^1–^ (blue) with theoretical extinction coefficients scaled down by a factor of 1.4.

Density functional theory (DFT) using the TPSSh functional^12^ was used to examine the (Cp′′3Th)^1–^ anion in **2** and **3**. Calculations using scalar-relativistic effective core potentials^13^ and triple-zeta valence basis sets, def-TZVP, for thorium^14^ predicted trigonal planar structures for Cp′′3Th and (Cp′′3Th)^1–^ that match the crystallographic data. The calculated Th–Cp′′(centroid) lengths of 2.538 Å for Cp′′3Th and 2.526 Å for (Cp′′3Th)^1–^ are similar to the experimentally determined distances of 2.52 Å. It is interesting to note that the calculations for the Th^3+^ complex show a slightly longer metal ligand distance than for the Th^2+^ complex. The calculations indicate a spin-paired ground state of 6d^2^ for (Cp′′3Th)^1–^ and a 6d^1^ ground state for Cp′′3Th; the latter is consistent with previous analyses of Cp′′3Th,^7g,m^ (C_5_Me_5_)_2_[^*i*^PrNC(Me)N^*i*^Pr]Th^7*n*^ and [K(DME)_2_]{[C_8_H_6_(Si^*t*^BuMe_2_)_2_]_2_Th}.^7*l*^ Gas-phase studies of Th^2+^ indicate a ground state of 5f^1^6d^1^, but the 6d^2^ configuration is just 63 cm^–1^ higher and the 5f^1^7s^1^ is 2527 cm^–1^ higher than the ground state.^15^ For (Cp_3_Th)^1–^ the triplet 5f^1^6d^1^ state is computed to be 9–14 kcal mol^–1^ higher in energy than the singlet 6d^2^ ground state.

The 6d^2^ singlet ground state can arise in this case due to stabilization of a d_*z*^2^_ orbital by the trigonal ligand environment as found in DFT calculations on (Cp′3Ln)^1–^ and (Cp′3U)^1–^ complexes^1c,d,4^ and noted earlier in the literature for tris(cyclopentadienyl) metal complexes.^7g,m,16^ Indeed, both the lowest unoccupied molecular orbital (LUMO) of Cp′′3Th and the highest occupied molecular orbital (HOMO) of (Cp′′3Th)^1–^ have d_*z*^2^_ character, Fig. 3. Complexes **2** and **3** provide the first examples of the 6d^2^ configuration since stable transition metal ions are only known with the 5d^*n*^ configurations of the third row transition metals. The 6d^2^ configuration is that predicted for ions like Rf^2+^ and Db^3+^.^17^

**Fig. 3:**
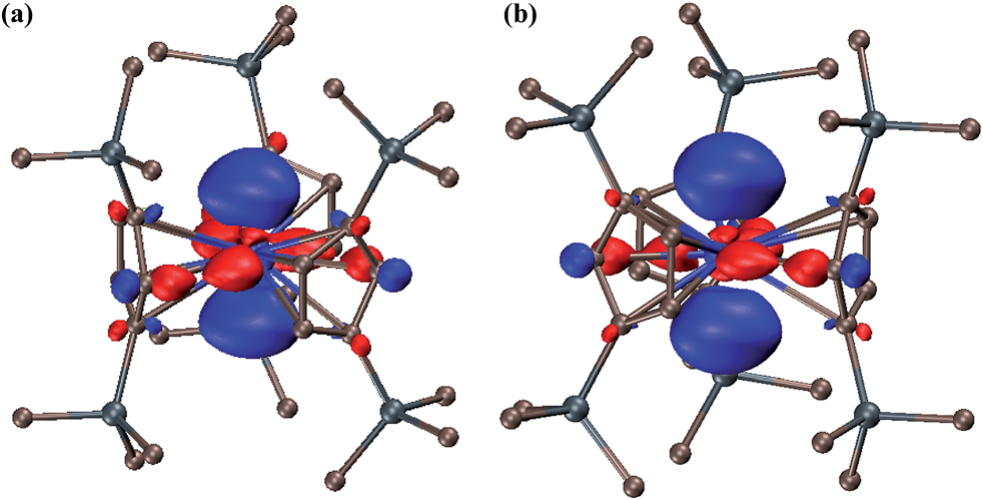
Contour plots of (a) the LUMO of Cp′′3Th and (b) the HOMO of the (Cp′′3Th)^1–^ anion in **3**. Contour value is 0.05.

Time-dependent density functional theory was used to simulate the UV-Vis spectra for the (Cp′′3Th)^1–^ anion as shown in Fig. 2 (see ESI† for a description of the predicted excitations). The maxima in the calculated spectra are lower in energy than those observed experimentally, but this is often the case with such calculations.^18^ Analysis of the calculated low energy peak shows that it arises from metal-to-metal transitions that have d → f and d → p character. The high energy peaks arise from metal-to-ligand charge transfer transitions similar to those found in the spectral analysis of (Cp′3Ln)^1–^ ^1b–d^ and (Cp′3U)^1–^.^4^ However, the d → f transitions found for (Cp′′3Th)^1–^ were not apparent in the analysis of the spectra of (Cp′3Ln)^1b–d^ and (Cp′3U)^1–^.^4^

The rate of decomposition of [K(18-crown-6)(THF)_2_][Cp′′3Th], **3**, at room temperature was studied by ^1^H NMR spectroscopy since monitoring by UV-Vis spectroscopy is complicated by the formation of highly colored Cp′′3Th, as identified by X-ray crystallography.^7*k*^ The rate of decomposition of **3** is much slower than that of the U^2+^ complex, [K(2.2.2-cryptand)][Cp′3U], which has a half-life of 1.5 h in THF at room temperature.^4^ Complex **3** decomposed only 8% after 8 days at 298 K and a sample kept in the dark showed even less decomposition. This suggests that the formally Th^2+^ species are significantly more stable than the other newly discovered +2 ions.^1d,4^

Complexes **2** and **3** were treated with H_2_ to determine if a Th^3+^ hydride complex such as “[K(2.2.2-cryptand)][Cp′′3ThH]” would form in analogy to the complex formed by reaction of [K(2.2.2-cryptand)][Cp′3U] with H_2_.^4^ Analogous chemistry is not observed with either H_2_ or KH. Complexes **2** and **3** react in solution within minutes with 1 atm of H_2_ and also over several hours at 60 psi in the solid state^19^ to make EPR active new crystalline complexes that appear to be bimetallic, but suitable models for the crystallographic data on the products have not been obtainable. The reactivity of **2** and **3** with H_2_ contrasts with that of the Th^3+^ complex, Cp′′3Th, which does not react under analogous conditions.

The (Cp′′3Th)^1–^ anion displays net two-electron reduction chemistry in its reaction with 1,3,5,7-cyclooctatetraene (C_8_H_8_). The Th^4+^ complex Cp′′2Th(C_8_H_8_), **4**, is formed as shown in eqn (4) and was characterized by X-ray crystallography, Fig. 4. The (C_8_H_8_)^2–^ ring in **4**, like that of (C_5_Me_4_H)_2_U(C_8_H_8_),^20^ displays considerable distortion from the normal planar geometry with several atoms 0.095 Å out of the best plane of the eight carbon atoms. This is reflected by a large range of Th–C(C_8_H_8_) distances: 2.736(4) to 2.841(4) Å. This 0.105 Å range is similar to the 0.123 Å range in (C_5_Me_4_H)_2_U(C_8_H_8_).^20^
4
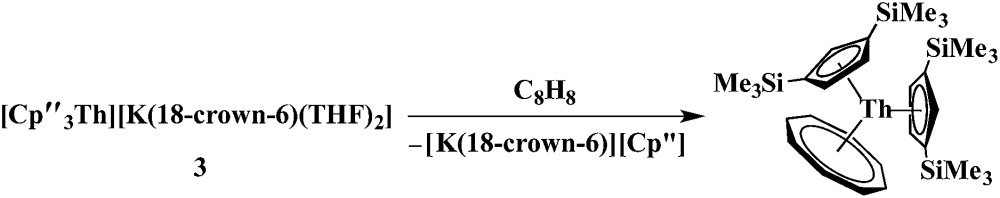



**Fig. 4:**
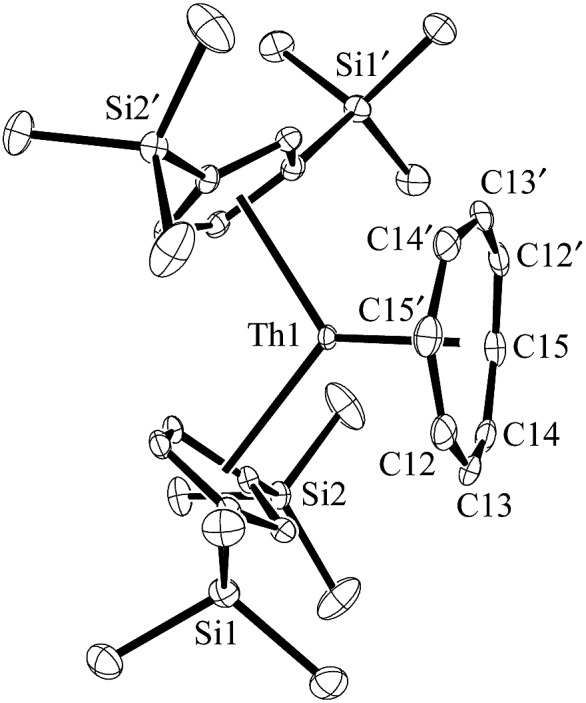
Molecular structure of Cp′′2Th(C_8_H_8_), **4**. Thermal ellipsoids are drawn at the 50% probability level and hydrogen atoms are omitted for clarity. Th–C(C_8_H_8_) distances (Å): Th–C12, 2.815(4); Th–C13, 2.841(4); Th–C14, 2.769(3); Th–C15, 2.736(4).

The isolation of the formally Th^2+^ ion in (Cp′′3Th)^1–^ is likely aided by the stabilization of the potassium counter-cation by the 18-crown-6 and 2.2.2-cryptand ligands. This was also observed with U^2+^ in the (Cp′3U)^1–^ anion^4^ and in the (Cp′3Ln)^1–^ complexes of the new Ln^2+^ ions.^1^ In the absence of these potassium-stabilizing chelates, isolation of Th^2+^ appears to be more difficult as described in a 2001 paper by Lappert and co-workers on the formation of Cp′′3Th by Na–K reduction of Cp′′3ThCl.^7*m*^ In that paper, Lappert reports that treatment of Cp′′3ThCl with excess Na–K alloy caused the initially blue solution (presumably Cp′′3Th) to change to dark green. They isolated a diamagnetic green compound they postulated to be “[K(THF)_*x*_][ThCp′′3] and/or ThCp′′2(THF)_*x*_” but they could not characterize it or obtain reproducible analytical results. Hence, the (Cp′′3Th)^1–^ anion was probably generated over 10 years ago, but could not be isolated in pure form as a simple [K(THF)_*x*_]^1+^ salt.

In summary, although it is difficult to obtain Th^3+^ complexes, further reduction is still possible with thorium: the +2 formal oxidation state of this metal is accessible in soluble molecular complexes. The Th^2+^ complexes provide the first examples of an isolable ion with a 6d^2^ electron configuration, the configuration possible for fourth row transition metal congeners of Hf^2+^ or Ta^3+^. The synthesis of these complexes demonstrates the power of specific ligand fields to generate new ground states with actinides. The identification of Th^2+^ is more evidence that the oxidation state diversity for the f elements is still increasing. Stabilization of higher-lying d orbitals by the ligand field appears to be a key factor in isolating these new ions and provides a new option in expanding the oxidation state chemistry of these elements. This approach should be pursued further as attempts are made to synthesize soluble molecular complexes of +1 ions of these metals.
