# Supplementary material for: Synthesis, structure, and reactivity of crystalline molecular complexes of the {[C5H3(SiMe3)2]3Th}1– anion containing thorium in the formal +2 oxidation state
Source: Chem Sci. 2014 Nov 3;6(1):517–21. doi: 10.1039/c4sc03033h (PMC5811171; doi:10.1039/c4sc03033h)
Supplement: Supplementary file 1 [file SC-006-C4SC03033H-s001.pdf]

Electronic Supplementary Material (ESI) for Chemical Science.
This journal is © The Royal Society of Chemistry 2014

Electronic Supporting Information

Synthesis, Structure, and Reactivity of Crystalline Molecular Complexes of the $\{[C_5H_3(SiMe_3)_2]_3Th\}^{1-}$ Anion Containing Thorium in the Formal +2 Oxidation State

Ryan R. Langeslay, Megan E. Fieser, Joseph W. Ziller, Filipp Furche, and William J. Evans*

Department of Chemistry, University of California, Irvine, California 92697-2025, United States

Email: wevans@uci.edu

*To whom correspondence should be addressed.

Table of Contents

Pages S3-S6	Experimental Details
Pages S6-S11	Computational Details
Pages S11-S21	X-ray Data Collection, Structure Solution and Refinement for Cp''_3Th, **1**.
Pages S21-S33	X-ray Data Collection, Structure Solution and Refinement for [K(2.2.2-cryptand)][Cp''_3Th], **2**.
Pages S34-S46	X-ray Data Collection, Structure Solution and Refinement for [K(18-crown-6)$(THF)_2$][Cp''_3Th], **3.**
Pages S46-S55	X-ray Data Collection, Structure Solution and Refinement for $Cp''_2Th(C_8H_8)$, **4.**
Pages S55-S56	Definitions and References

EXPERIMENTAL DETAILS

All manipulations and syntheses described below were conducted with the rigorous exclusion of air and water using standard Schlenk line and glovebox techniques under an argon or dinitrogen atmosphere. Solvents were sparged with UHP argon and dried by passage through columns containing Q-5 and molecular sieves prior to use. Deuterated NMR solvents were dried over NaK alloy, degassed by three freeze-pump-thaw cycles, and vacuum transferred before use. ^{1}H NMR spectra were recorded on Bruker DR400, GN500, or CRYO500 MHz spectrometers (^{13}C NMR spectra on the 500 MHz spectrometer operating at 125 MHz) at 298 K unless otherwise stated and referenced internally to residual protio-solvent resonances. IR samples were prepared as KBr pellets and analyzed using a Varian 1000 FT-IR Scimitar Series spectrometer. Elemental analyses were conducted on a Perkin-Elmer 2400 Series II CHNS elemental analyzer. $ThBr_4(THF)_4$,[1] KC_8,[2] and $K[C_5H_4(SiMe_3)]$[3] were prepared according to the literature. $K[C_5H_3(SiMe_3)_2]$ (KCp″) was prepared from KCp′ in an analogous procedure to the preparation of KCp′ from KCp.[3]

$[C_5H_3(SiMe_3)_2]_3ThBr$. Solid $K[C_5H_3(SiMe_3)_2]$ (1.120 g, 4.506 mmol) was added to a stirred colorless solution of $ThBr_4(THF)_4$ (1.197 g, 1.273 mmol) in Et_2O (100 mL). After 24 h, the solvent was removed from the white slurry under vacuum and the white solids were extracted with hexane (40 mL). The extract was concentrated to 6 mL, heated and stirred to dissolve all solids, and placed at −30 ºC for 24 h. This yielded colorless crystals that were analyzed by X-ray crystallography. However, a suitable model for the crystallographic data has not been obtainable. A second crop of crystals was obtained and combined with the first (0.819 g, 60%). ^{1}H NMR (C_6D_6): δ 6.90 [m, 3H, $C_5H_3(SiMe_3)$], 0.44 [s, 54H, $C_5H_3(SiMe_3)$]. ^{13}C NMR (C_6D_6): δ 136.22 [$C_5H_3(SiMe_3)$], 130.09 [$C_5H_3(SiMe_3)$], 128.37 [$C_5H_3(SiMe_3)$], 1.45 [$C_5H_3(SiMe_3)$]. IR:

3051w, 2953m, 2896m, 1440w, 1405w, 1318w, 1247s, 1203w, 1083s, 1057w, 924s, 834s, 791s, 753s, 689m, 637m, 613m cm^{-1}. Anal. Calcd for $C_{33}H_{63}BrSi_6Th$: C, 42.15, H, 6.57. Found: C, 41.83, H, 6.87.

$[C_5H_3(SiMe_3)_2]_3Th$, 1. This is a variation of the literature preparation using a bromide precursor instead of a chloride precursor.[4] When KC_8 (0.127 g, 0.940 mmol) was slowly tapped into a stirred colorless solution of $[C_5H_3(SiMe_3)_2]_3ThBr$ (0.516 g, 0.549 mmol) in THF (10 mL), the solution immediately turned dark blue. The mixture was allowed to stir for 1 h before being centrifuged and filtered to remove graphite, KBr, and excess KC_8. The solids were washed twice with 5 mL of THF, the wash solutions were filtered and combined with the initial filtrate, and solvent was removed under vacuum to yield dark blue solids. The solids were extracted with hexane (18 mL) and the solution was dried under vacuum to yield **1** as a dark blue solid (0.419 g, 0.487 mmol, 89%). The complex was identified by X-ray crystallography.[4]

$[K(2.2.2\text{-cryptand})][(C_5H_3(SiMe_3)_2)_3Th]$, 2. 2.2.2-Cryptand (92 mg, 0.24 mmol) was added to a stirred dark blue solution of **1** (210 mg, 0.244 mmol) in THF (7 mL). KC_8 (50 mg, 0.37 mmol) was tapped into the reaction vessel and the mixture was allowed to stir for 4 min. The mixture was centrifuged and filtered to remove graphite and excess KC_8. The solids were washed with THF (5 mL) and the resulting aqua green solution was filtered and combined with the initial green filtrate. Solvent was removed under vacuum to yield dichroic dark blue/red solids. The product was washed with hexane (5 mL) to remove any unreacted starting material and dried to yield **2** (231 mg, 74%). Dichroic dark blue/red X-ray quality crystals were grown from a cold Et_2O solution (3 mL) of **2** (231 mg) layered with cold hexane (17 mL). ^{1}H NMR (THF-d_8): δ 4.44 [s, 6H, $C_5H_3(SiMe_3)_2$] 3.59 [s, 12H, OCH_2CH_2O], 3.55 [t, $^1J_{HH}$ = 5 Hz, 12H, NCH_2CH_2O], 2.57 [t, $^1J_{HH}$ = 5 Hz, 12H, NCH_2CH_2O], 0.21 [s 54H, $C_5H_3(SiMe_3)$]. ^{13}C NMR

(THF-d_8): δ 115.54 [$C_5H_3(SiMe_3)_2$], 71.32 [OCH_2CH_2O], 68.45 [NCH_2CH_2O], 54.75 [NCH_2CH_2O], 2.38 [$C_5H_3(SiMe_3)_2$]. IR: 3051w, 3035w, 3014w, 2949m, 2889m, 2814m, 1476w, 1460w, 1445w, 1418w, 1397w, 1361m, 1298w, 1238m, 1174m, 1134m, 1107s, 1075s, 952m, 934m, 911m, 829s, 785m, 746m, 675w, 627m cm^{-1}. Anal. Calcd for $C_{51}H_{99}KN_2O_6Si_6Th$: C, 48.01, H, 7.82, N, 2.20. Found C, 47.81, H, 8.18, N, 2.21.

[K(18-crown-6)(THF)$_2$][($C_5H_3(SiMe_3)_2)_3$Th], 3. Complex **3** was obtained by following an analogous procedure to that for compound **2** but with 18-crown-6 (35 mg, 0.13 mmol) instead of 2.2.2-cryptand and a solution of $[C_5H_3(SiMe_3)_2]_3$Th (113 mg, 0.130 mmol) in 7 mL of THF and KC_8 (27 mg, 0.20 mmol). This generated **3** (124 mg, 81%) as dichroic dark blue/red X-ray quality crystals. ^{1}H NMR (THF-d_8): δ 4.58 [s, 6H, $C_5H_3(SiMe_3)_2$], 3.61 [s, 24H, $C_{12}H_{24}O_6$], 0.33 [s, 54H, $C_5H_3(SiMe_3)_2$]. ^{13}C NMR (THF-d_8): δ 115.61 [$C_5H_3(SiMe_3)_2$], 71.15 [OCH_2CH_2O], 2.28 [$C_5H_3(SiMe_3)_2$]. IR: 3052w, 2951m, 2894m. 2363w, 2338w, 1638w, 1473w, 1453w, 1397m, 1352m, 1308w, 1283w, 1242s, 1167m, 1110s, 1075s, 1055m, 964m, 913m, 831s, 786m, 750w, 714w, 687w cm^{-1}. Anal. Calcd for $C_{43}H_{83}KO_6Si_6Th$: C, 46.44, H, 7.54. Found: C, 46.27, H, 7.79.

($C_5H_3(SiMe_3)_2)_2$Th(C_8H_8), 4. Cyclooctatetraene (25 μL, 0.22 mmol) was added to a stirred dark green solution of **3** (147 mg, 0.112 mmol) in THF (8 mL). The solution was allowed to stir overnight, by which time it had turned yellow. The solution was dried under vacuum to yield a yellow solid which was then extracted into 20 mL of hexane, filtered, and dried under vacuum to yield **4** as a yellow solid (71 mg, 75%). Yellow X-ray quality crystals were grown from a pentane solution at −35 ºC. ^{1}H NMR (C_6D_6): δ 6.79 [t, $^2J_{HH}$ = 2 Hz, 2H, $C_5H_3(SiMe_3)_2$], 6.40 [s, 8H, C_8H_8], 6.27 [d, $^2J_{HH}$ = 2 Hz, 4H, $C_5H_3(SiMe_3)_2$], 0.33 [s, 36H, $C_5H_3(SiMe_3)_2$]. ^{13}C NMR (C_6D_6): δ 132.09 [$C_5H_3(SiMe_3)_2$], 131.75 [$C_5H_3(SiMe_3)_2$], 128.36 [$C_5H_3(SiMe_3)_2$], 100.62

(C_8H_8), 1.20 [$C_5H_3(SiMe_3)_2$]. IR: 3088w, 3043w, 2956m, 2895w, 1442w, 1401w, 1323w, 1244s, 1211w, 1084s, 923m, 832s, 754m, 719s, 691m, 638m, 620m cm^{-1}. Anal. Calcd for $C_{30}H_{50}Si_4Th$: C, 47.72, H, 6.67. Found: C, 47.36, H, 6.63.

Computational Details.

Initial gas-phase structural optimizations were performed starting from the crystal data of Cp''_3Th, **1**, and [K(18-crown-6)(THF)$_2$][Cp''_3Th], **3**, using the hybrid meta-GGA functional, TPSSh.[5] Countercations were removed from the crystal structure of [K(18-crown-6)(THF)$_2$][Cp''_3Th] before the start of the optimizations. Double-zeta quality split-valence basis sets with polarization functions, [def2-SV(P)],[6] were used for all non-hydrogen, light atoms. Scalar-relativistic effective core potentials (ECPs)[7] and triple-zeta valence basis sets, def-TZVP,[8] with the two tight g functions removed, were used for Th. Vibrational frequencies[9] were computed for the optimized structure of $(Cp''_3Th)^-$ and the structure was confirmed to be a potential energy minimum by the absence of imaginary modes.

In order to account for solvation effects, a second structural optimization was carried out using the continuum solvation model (COSMO).[10] The dielectric constant of THF ($\varepsilon = 7.520$)[11] was used for the reported COSMO calculations since this was the most polar solvent used in the synthesis. Solvent effects have been shown to be important for Ln^{2+} systems because they screen the additional negative charge. The changes in bond length between the SV(P) optimized structures and those from a third optimization using TZVP basis sets for light atoms were typically smaller than 0.02 Å.[12] The COSMO/TZVP optimized coordinates of Cp''_3Th and [K(18-crown-6)][Cp''_3Th] in xyz format are provided in the Electronic Supporting Information. All results were computed in C_1 symmetry and ground state energies were converged to 10^{-7} a.u.

using fine quadrature grids (at least size m4). Molecular orbitals were plotted with a contour value of 0.05. All calculations were carried out using the TURBOMOLE version 6.5 program package.[13]

To corroborate the assignment of the ground state configuration, the singlet ground state and the first triplet state of the $(Cp_3Th)^{1-}$ model compound were optimized using the TPSS[5] functional and TZVP basis sets for light atoms, while larger TZVPP basis sets were employed for Th; solvation effects were included at the COSMO level as described above. These calculations yielded a 9 kcal/mol energy difference between the singlet $6d^2$ ground state and the triplet $5f^16d^1$ state. Additional single-point energy calculations using the random phase approximation[14] produced an energy difference of 14 kcal/mol in favor of the singlet $6d^2$ state, confirming the TPSS and TPSSh results.

Time dependent DFT (TDDFT) excitation energy calculations[15] of the lowest 60 spin-unrestricted excitations of Cp''_3Th, **1**, and the lowest 60 excitations from self-consistent orbitals for $(Cp''_3Th)^-$ were carried out on the solvent optimized structures using the recently developed gauge-invariant implementation[16] of TPSSh and split-valence basis sets, Figures S1 and S2. Diffuse functions were also added to the basis set (def2-SVPD)[17] for light atoms, while small core ECPs and TZVP basis sets were used for thorium. To simulate the UV-Vis spectra, a normalized Gaussian scaled by the predicted oscillator strength was centered at each molecular excitation, and a root mean square width of 0.14 eV was chosen to fit experiment.[18] Representative excitations (in nm) and oscillator strengths in the length gauge (in a.u.) from each band, and dominant single-particle contributions for each transition are reported in Table S1.

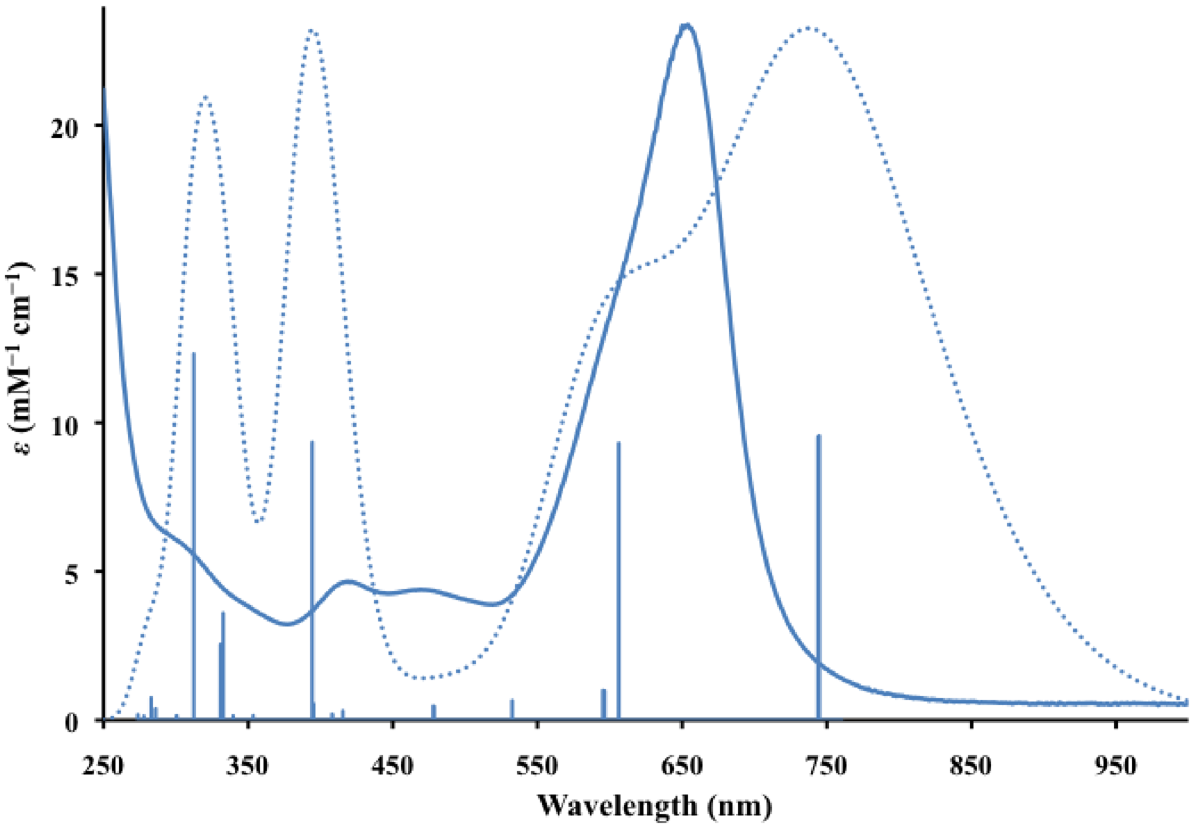

Figure S1. Experimental (solid) and calculated (dotted) UV-vis spectra of [K(18-crown-6)][Cp″$_3$Th] in THF at 298 K, with pertinent theoretical excitations shown as vertical lines and theoretical extinction coefficients scaled down by a factor of 1.4.

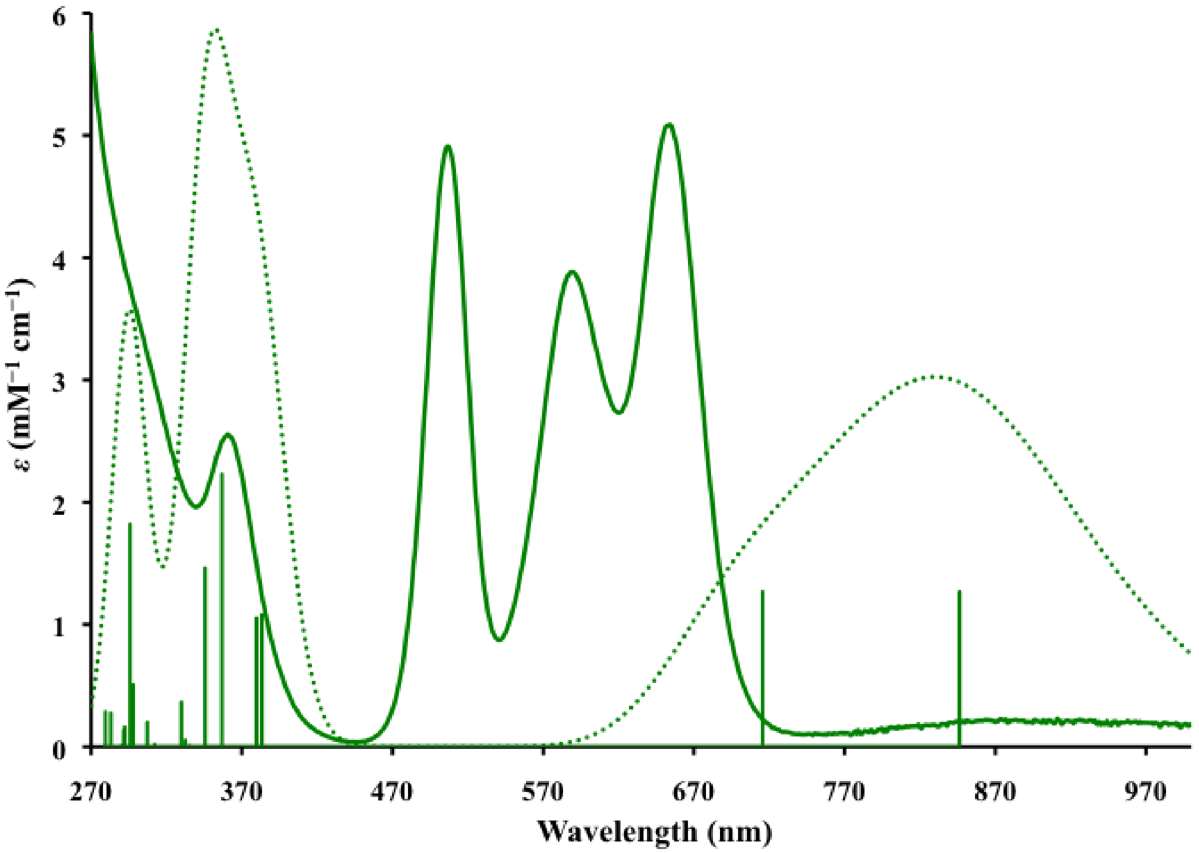

Figure S2. Experimental (solid) and calculated (dotted) UV-vis spectra of Cp''_3Th in THF at 298 K, with pertinent theoretical excitations shown as vertical lines and theoretical extinction coefficients scaled down by a factor of 1.5.

Table S1. Electronic excitation summary for Cp″$_3$Th, **1**, and (Cp″$_3$Th)$^-$ (anion of **3**) computed using TPSSh and SVPD basis sets for light atoms, and corresponding ECPs/basis sets for the heavy metals as described in the text. Only excitations that have the largest oscillator strength (in the length gauge) from each band are reported. The 188 α singly occupied orbital is the HOMO for **1**, while the 188 α doubly occupied orbital is the HOMO for (Cp″$_3$Th)$^-$.

**Compound**	**Excitation #**	**Wavelength (nm)**	**Oscillator Strength (len)**	**Dominant Contributions**		
				**occupied**	**virtual**	**% weight**
Cp″$_3$Th	3	846.3	0.025	188 α	191 α	93.6
	4	846.0	0.025	188 α	192 α	93.6
	6	715.7	0.025	188 α	194 α	97.9
	11	383.5	0.022	188 α	197 α	91.1
	12	383.3	0.022	188 α	198 α	91.0
	13	379.8	0.021	188 α	199 α	90.4
	15	356.8	0.045	186 β	188 β	90.6
	18	345.7	0.029	188 α	207 α	71.5
				188 α	203 α	11.6
	19	345.6	0.029	188 α	208 α	72.8
				188 α	202 α	10.3
	39	298.1	0.010	183 β	190 β	13.6
				182 β	189 β	13.5
	41	295.9	0.036	188 α	214 α	29.6
				188 α	217 α	21.0
				188 α	223 α	11.7
(Cp″$_3$Th)$^-$	4	744.8	0.096	188 α	191α	94.7
	5	743.8	0.095	188 α	192 α	94.7
	7	606.2	0.093	188 α	195 α	98.5
	9	595.3	0.010	188 α	197 α	98.4
	24	394.3	0.094	188 α	212 α	82.1
	25	394.2	0.093	188 α	213 α	81.9
	35	332.7	0.036	188 α	225 α	94.2
	36	332.6	0.036	188 α	226 α	94.5
	38	330.8	0.026	188 α	224 α	70.0
				188 α	223 α	25.4
	39	312.3	0.123	188 α	227 α	93.8

Transitions were analyzed with a Mulliken population analysis (MPA).[19] Between 500 and 800 nm the predicted excitations are primarily metal-to-metal with strong d/s→p/f character and minor ligand contributions. Excitations around 750 nm have mostly d/s→f character, while the excitations localized near 600 nm have mostly d/s→p character. Two large groups of excitations are predicted in the 300-400 nm range, which consist of d/s→π* excitations. As the wavelength decreases, the transitions go to orbitals with more ligand character, similar to that seen for the lanthanides and uranium. Predicted excitations for Cp''_3Th are very similar to those for $(Cp''_3Th)^-$, although the oscillator strengths for the excitations are much weaker than for $(Cp''_3Th)^-$, which is consistent with intensities of the experimental spectra. Between 370 and 900 nm, the predicted excitations are primarily metal-to-metal, with d/s→f character between 700 and 900 nm and d/s→p character between 370 and 500 nm. Below 370 nm, the transitions consist of d/s→π* excitations.

X-ray Data Collection, Structure Solution and Refinement for 1.

A purple crystal of approximate dimensions 0.136 x 0.219 x 0.344 mm was mounted on a glass fiber and transferred to a Bruker SMART APEX II diffractometer. The APEX2[20] program package was used to determine the unit-cell parameters and for data collection (15 sec/frame scan time for a sphere of diffraction data). The raw frame data was processed using SAINT[21] and SADABS[22] to yield the reflection data file. Subsequent calculations were carried out using the SHELXTL[23] program. The diffraction symmetry was 2/*m* and the systematic absences were consistent with the monoclinic space group $P2_1/c$ that was later determined to be correct.

The structure was solved using the coordinates of the room temperature data set[24] and refined on F^2 by full-matrix least-squares techniques. The analytical scattering factors[25] for neutral atoms were used throughout the analysis. Hydrogen atoms were included using a riding model.

At convergence, wR2 = 0.0478 and Goof = 1.038 for 379 variables refined against 9378 data (0.78Å), R1 = 0.0208 for those _ data with I > 2.0σ(I).

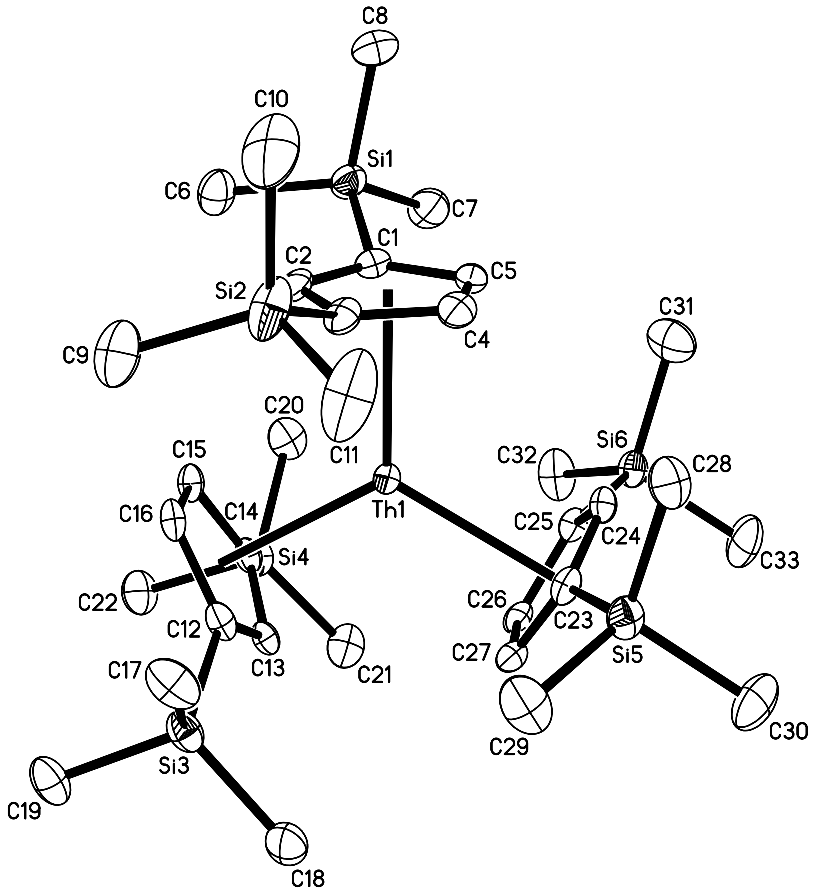

Figure S3, molecular structure of **1**, with thermal ellipsoids drawn at the 50% probability level and hydrogen atoms omitted for clarity.

Table S2. Crystal data and structure refinement for **1.**

Table 1. Crystal data and structure refinement for rrl24.

Identification code	rrl24 (Ryan Langeslay)
Empirical formula	$C_{33} H_{63} Si_6 Th$
Formula weight	860.41
Temperature	133(2) K
Wavelength	0.71073 Å
Crystal system	Monoclinic

Space group	$P2_1/c$	
Unit cell dimensions	a = 17.5470(10) Å	α= 90°.
	b = 13.6955(8) Å	β= 112.5954(7)°.
	c = 19.1293(11) Å	γ = 90°.
Volume	4244.2(4) Å^3	
Z	4	
Density (calculated)	1.347 Mg/m^3	
Absorption coefficient	3.702 mm^{-1}	
F(000)	1740	
Crystal color	purple	
Crystal size	0.344 x 0.219 x 0.136 mm^3	
Theta range for data collection	1.257 to 27.103°	
Index ranges	$-22 \leq h \leq 22$, $-17 \leq k \leq 17$, $-24 \leq l \leq 24$	
Reflections collected	47736	
Independent reflections	9378 [R(int) = 0.0328]	
Completeness to theta = 25.242°	100.0 %	
Absorption correction	Numerical	
Max. and min. transmission	0.7193 and 0.4749	
Refinement method	Full-matrix least-squares on F^2	
Data / restraints / parameters	9378 / 0 / 379	
Goodness-of-fit on F^2	1.038	
Final R indices [I>2sigma(I) = 8179 data]	R1 = 0.0208, wR2 = 0.0455	
R indices (all data, 0.78Å)	R1 = 0.0275, wR2 = 0.0478	
Largest diff. peak and hole	1.272 and -0.705 e.Å^{-3}	

Table S3. Bond lengths [Å] and angles [°] for **1.**

Th(1)-Cnt1	2.517
Th(1)-Cnt2	2.521
Th(1)-Cnt3	2.521
Th(1)-C(26)	2.772(2)
Th(1)-C(4)	2.779(2)
Th(1)-C(2)	2.782(2)
Th(1)-C(27)	2.787(2)
Th(1)-C(13)	2.788(2)

Th(1)-C(3)	2.788(2)
Th(1)-C(16)	2.790(2)
Th(1)-C(5)	2.794(2)
Th(1)-C(15)	2.794(2)
Th(1)-C(24)	2.799(2)
Th(1)-C(25)	2.801(2)
Th(1)-C(12)	2.806(2)
Th(1)-C(14)	2.806(2)
Th(1)-C(1)	2.814(2)
Th(1)-C(23)	2.821(2)
Si(1)-C(6)	1.865(3)
Si(1)-C(1)	1.866(3)
Si(1)-C(7)	1.866(3)
Si(1)-C(8)	1.875(3)
Si(2)-C(3)	1.857(3)
Si(2)-C(11)	1.860(4)
Si(2)-C(9)	1.861(3)
Si(2)-C(10)	1.864(4)
Si(3)-C(12)	1.863(2)
Si(3)-C(18)	1.867(3)
Si(3)-C(19)	1.868(3)
Si(3)-C(17)	1.870(3)
Si(4)-C(14)	1.863(2)
Si(4)-C(21)	1.864(3)
Si(4)-C(20)	1.864(3)
Si(4)-C(22)	1.874(3)
Si(5)-C(30)	1.864(3)
Si(5)-C(29)	1.866(3)
Si(5)-C(28)	1.868(3)
Si(5)-C(23)	1.870(2)
Si(6)-C(32)	1.858(3)
Si(6)-C(31)	1.860(3)
Si(6)-C(25)	1.863(2)
Si(6)-C(33)	1.866(3)
C(1)-C(2)	1.417(3)
C(1)-C(5)	1.431(3)

C(2)-C(3)	1.426(3)
C(3)-C(4)	1.430(3)
C(4)-C(5)	1.397(4)
C(12)-C(13)	1.424(3)
C(12)-C(16)	1.431(4)
C(13)-C(14)	1.422(3)
C(14)-C(15)	1.438(3)
C(15)-C(16)	1.403(3)
C(23)-C(24)	1.425(3)
C(23)-C(27)	1.432(3)
C(24)-C(25)	1.429(3)
C(25)-C(26)	1.428(3)
C(26)-C(27)	1.401(3)

Cnt1-Th(1)-Cnt2	120.1
Cnt1-Th(1)-Cnt3	119.8
Cnt2-Th(1)-Cnt3	120.1
C(26)-Th(1)-C(4)	122.81(7)
C(26)-Th(1)-C(2)	160.11(7)
C(4)-Th(1)-C(2)	47.99(7)
C(26)-Th(1)-C(27)	29.20(7)
C(4)-Th(1)-C(27)	113.15(7)
C(2)-Th(1)-C(27)	159.69(7)
C(26)-Th(1)-C(13)	74.02(7)
C(4)-Th(1)-C(13)	158.89(7)
C(2)-Th(1)-C(13)	120.14(7)
C(27)-Th(1)-C(13)	74.71(7)
C(26)-Th(1)-C(3)	150.55(7)
C(4)-Th(1)-C(3)	29.77(7)
C(2)-Th(1)-C(3)	29.66(7)
C(27)-Th(1)-C(3)	130.42(7)
C(13)-Th(1)-C(3)	130.28(7)
C(26)-Th(1)-C(16)	121.65(7)
C(4)-Th(1)-C(16)	113.00(7)
C(2)-Th(1)-C(16)	74.85(7)
C(27)-Th(1)-C(16)	113.55(7)

C(13)-Th(1)-C(16)	47.90(7)
C(3)-Th(1)-C(16)	83.24(7)
C(26)-Th(1)-C(5)	114.47(7)
C(4)-Th(1)-C(5)	29.04(7)
C(2)-Th(1)-C(5)	47.79(7)
C(27)-Th(1)-C(5)	121.19(7)
C(13)-Th(1)-C(5)	161.33(7)
C(3)-Th(1)-C(5)	48.86(7)
C(16)-Th(1)-C(5)	122.62(7)
C(26)-Th(1)-C(15)	114.30(7)
C(4)-Th(1)-C(15)	121.63(7)
C(2)-Th(1)-C(15)	74.07(7)
C(27)-Th(1)-C(15)	122.64(7)
C(13)-Th(1)-C(15)	48.00(7)
C(3)-Th(1)-C(15)	95.09(7)
C(16)-Th(1)-C(15)	29.09(7)
C(5)-Th(1)-C(15)	114.91(7)
C(26)-Th(1)-C(24)	47.86(7)
C(4)-Th(1)-C(24)	74.98(7)
C(2)-Th(1)-C(24)	119.92(7)
C(27)-Th(1)-C(24)	47.88(7)
C(13)-Th(1)-C(24)	119.93(7)
C(3)-Th(1)-C(24)	103.34(7)
C(16)-Th(1)-C(24)	159.82(7)
C(5)-Th(1)-C(24)	73.72(7)
C(15)-Th(1)-C(24)	160.22(7)
C(26)-Th(1)-C(25)	29.68(7)
C(4)-Th(1)-C(25)	97.76(7)
C(2)-Th(1)-C(25)	131.18(7)
C(27)-Th(1)-C(25)	48.99(7)
C(13)-Th(1)-C(25)	101.63(7)
C(3)-Th(1)-C(25)	127.53(7)
C(16)-Th(1)-C(25)	149.23(7)
C(5)-Th(1)-C(25)	84.86(7)
C(15)-Th(1)-C(25)	130.85(7)
C(24)-Th(1)-C(25)	29.56(7)

C(26)-Th(1)-C(12)	95.60(7)
C(4)-Th(1)-C(12)	129.59(8)
C(2)-Th(1)-C(12)	102.88(7)
C(27)-Th(1)-C(12)	83.92(7)
C(13)-Th(1)-C(12)	29.50(7)
C(3)-Th(1)-C(12)	102.91(7)
C(16)-Th(1)-C(12)	29.62(7)
C(5)-Th(1)-C(12)	149.83(7)
C(15)-Th(1)-C(12)	48.85(7)
C(24)-Th(1)-C(12)	131.05(7)
C(25)-Th(1)-C(12)	125.26(7)
C(26)-Th(1)-C(14)	84.56(7)
C(4)-Th(1)-C(14)	149.69(7)
C(2)-Th(1)-C(14)	101.82(7)
C(27)-Th(1)-C(14)	97.12(7)
C(13)-Th(1)-C(14)	29.46(7)
C(3)-Th(1)-C(14)	124.86(7)
C(16)-Th(1)-C(14)	48.81(7)
C(5)-Th(1)-C(14)	132.13(7)
C(15)-Th(1)-C(14)	29.77(7)
C(24)-Th(1)-C(14)	131.26(7)
C(25)-Th(1)-C(14)	103.48(7)
C(12)-Th(1)-C(14)	49.54(7)
C(26)-Th(1)-C(1)	130.92(7)
C(4)-Th(1)-C(1)	48.74(7)
C(2)-Th(1)-C(1)	29.32(7)
C(27)-Th(1)-C(1)	148.72(7)
C(13)-Th(1)-C(1)	132.32(7)
C(3)-Th(1)-C(1)	49.58(7)
C(16)-Th(1)-C(1)	97.71(7)
C(5)-Th(1)-C(1)	29.56(7)
C(15)-Th(1)-C(1)	85.42(7)
C(24)-Th(1)-C(1)	101.02(7)
C(25)-Th(1)-C(1)	103.31(7)
C(12)-Th(1)-C(1)	127.33(7)
C(14)-Th(1)-C(1)	104.64(7)

C(26)-Th(1)-C(23)	48.74(7)
C(4)-Th(1)-C(23)	83.57(7)
C(2)-Th(1)-C(23)	131.00(7)
C(27)-Th(1)-C(23)	29.59(7)
C(13)-Th(1)-C(23)	102.79(7)
C(3)-Th(1)-C(23)	103.93(7)
C(16)-Th(1)-C(23)	130.75(7)
C(5)-Th(1)-C(23)	94.76(7)
C(15)-Th(1)-C(23)	150.29(7)
C(24)-Th(1)-C(23)	29.36(7)
C(25)-Th(1)-C(23)	49.56(7)
C(12)-Th(1)-C(23)	103.89(7)
C(14)-Th(1)-C(23)	126.71(7)
C(1)-Th(1)-C(23)	124.29(7)
C(6)-Si(1)-C(1)	109.23(12)
C(6)-Si(1)-C(7)	110.92(14)
C(1)-Si(1)-C(7)	114.42(13)
C(6)-Si(1)-C(8)	109.36(14)
C(1)-Si(1)-C(8)	105.17(13)
C(7)-Si(1)-C(8)	107.49(14)
C(3)-Si(2)-C(11)	111.47(13)
C(3)-Si(2)-C(9)	112.23(12)
C(11)-Si(2)-C(9)	111.20(17)
C(3)-Si(2)-C(10)	105.05(16)
C(11)-Si(2)-C(10)	109.2(2)
C(9)-Si(2)-C(10)	107.36(16)
C(12)-Si(3)-C(18)	110.36(12)
C(12)-Si(3)-C(19)	105.15(12)
C(18)-Si(3)-C(19)	109.63(13)
C(12)-Si(3)-C(17)	112.72(12)
C(18)-Si(3)-C(17)	109.74(14)
C(19)-Si(3)-C(17)	109.12(13)
C(14)-Si(4)-C(21)	110.38(12)
C(14)-Si(4)-C(20)	109.94(12)
C(21)-Si(4)-C(20)	111.58(13)
C(14)-Si(4)-C(22)	106.79(12)

C(21)-Si(4)-C(22)	108.88(13)
C(20)-Si(4)-C(22)	109.14(13)
C(30)-Si(5)-C(29)	108.34(15)
C(30)-Si(5)-C(28)	107.51(14)
C(29)-Si(5)-C(28)	110.94(14)
C(30)-Si(5)-C(23)	107.11(12)
C(29)-Si(5)-C(23)	112.50(12)
C(28)-Si(5)-C(23)	110.22(12)
C(32)-Si(6)-C(31)	110.42(15)
C(32)-Si(6)-C(25)	113.30(12)
C(31)-Si(6)-C(25)	110.56(12)
C(32)-Si(6)-C(33)	108.07(15)
C(31)-Si(6)-C(33)	107.51(15)
C(25)-Si(6)-C(33)	106.72(13)
C(2)-C(1)-C(5)	105.0(2)
C(2)-C(1)-Si(1)	123.19(19)
C(5)-C(1)-Si(1)	126.41(19)
C(2)-C(1)-Th(1)	74.09(14)
C(5)-C(1)-Th(1)	74.43(14)
Si(1)-C(1)-Th(1)	136.82(12)
C(1)-C(2)-C(3)	111.5(2)
C(1)-C(2)-Th(1)	76.59(14)
C(3)-C(2)-Th(1)	75.40(14)
C(2)-C(3)-C(4)	104.7(2)
C(2)-C(3)-Si(2)	124.77(19)
C(4)-C(3)-Si(2)	126.23(19)
C(2)-C(3)-Th(1)	74.93(13)
C(4)-C(3)-Th(1)	74.75(13)
Si(2)-C(3)-Th(1)	133.41(12)
C(5)-C(4)-C(3)	109.5(2)
C(5)-C(4)-Th(1)	76.07(14)
C(3)-C(4)-Th(1)	75.48(14)
C(4)-C(5)-C(1)	109.4(2)
C(4)-C(5)-Th(1)	74.89(14)
C(1)-C(5)-Th(1)	76.01(14)
C(13)-C(12)-C(16)	105.0(2)

C(13)-C(12)-Si(3)	124.54(19)
C(16)-C(12)-Si(3)	126.41(19)
C(13)-C(12)-Th(1)	74.55(13)
C(16)-C(12)-Th(1)	74.60(13)
Si(3)-C(12)-Th(1)	133.54(11)
C(14)-C(13)-C(12)	111.4(2)
C(14)-C(13)-Th(1)	75.99(13)
C(12)-C(13)-Th(1)	75.96(13)
C(13)-C(14)-C(15)	105.1(2)
C(13)-C(14)-Si(4)	126.97(19)
C(15)-C(14)-Si(4)	125.18(18)
C(13)-C(14)-Th(1)	74.55(13)
C(15)-C(14)-Th(1)	74.66(13)
Si(4)-C(14)-Th(1)	130.41(11)
C(16)-C(15)-C(14)	109.0(2)
C(16)-C(15)-Th(1)	75.30(13)
C(14)-C(15)-Th(1)	75.57(13)
C(15)-C(16)-C(12)	109.6(2)
C(15)-C(16)-Th(1)	75.61(13)
C(12)-C(16)-Th(1)	75.78(13)
C(24)-C(23)-C(27)	105.0(2)
C(24)-C(23)-Si(5)	124.38(18)
C(27)-C(23)-Si(5)	126.93(19)
C(24)-C(23)-Th(1)	74.48(13)
C(27)-C(23)-Th(1)	73.89(13)
Si(5)-C(23)-Th(1)	133.31(11)
C(23)-C(24)-C(25)	111.4(2)
C(23)-C(24)-Th(1)	76.16(13)
C(25)-C(24)-Th(1)	75.27(13)
C(26)-C(25)-C(24)	104.6(2)
C(26)-C(25)-Si(6)	127.82(18)
C(24)-C(25)-Si(6)	124.05(18)
C(26)-C(25)-Th(1)	74.04(13)
C(24)-C(25)-Th(1)	75.17(13)
Si(6)-C(25)-Th(1)	132.00(11)
C(27)-C(26)-C(25)	109.9(2)

C(27)-C(26)-Th(1)	75.99(13)
C(25)-C(26)-Th(1)	76.27(13)
C(26)-C(27)-C(23)	109.1(2)
C(26)-C(27)-Th(1)	74.81(13)
C(23)-C(27)-Th(1)	76.52(13)

X-ray Data Collection, Structure Solution and Refinement for 2.

A red crystal of approximate dimensions 0.307 x 0.138 x 0.056 mm was mounted on a glass fiber and transferred to a Bruker SMART APEX II diffractometer. The APEX2[26] program package was used to determine the unit-cell parameters and for data collection (60 sec/frame scan time for a sphere of diffraction data). The raw frame data was processed using SAINT[21] and SADABS[22] to yield the reflection data file. Subsequent calculations were carried out using the SHELXTL[23] program. There were no systematic absences nor any diffraction symmetry other than the Friedel condition. The centrosymmetric triclinic space group $P\bar{1}$ was assigned and later determined to be correct.

The structure was solved by direct methods and refined on F^2 by full-matrix least-squares techniques. The analytical scattering factors[25] for neutral atoms were used throughout the analysis. Hydrogen atoms were included using a riding model.

At convergence, wR2 = 0.0621 and Goof = 1.010 for 622 variables refined against 15230 data (0.74 Å), R1 = 0.0287 for those 13490 data with $I > 2.0\sigma(I)$.

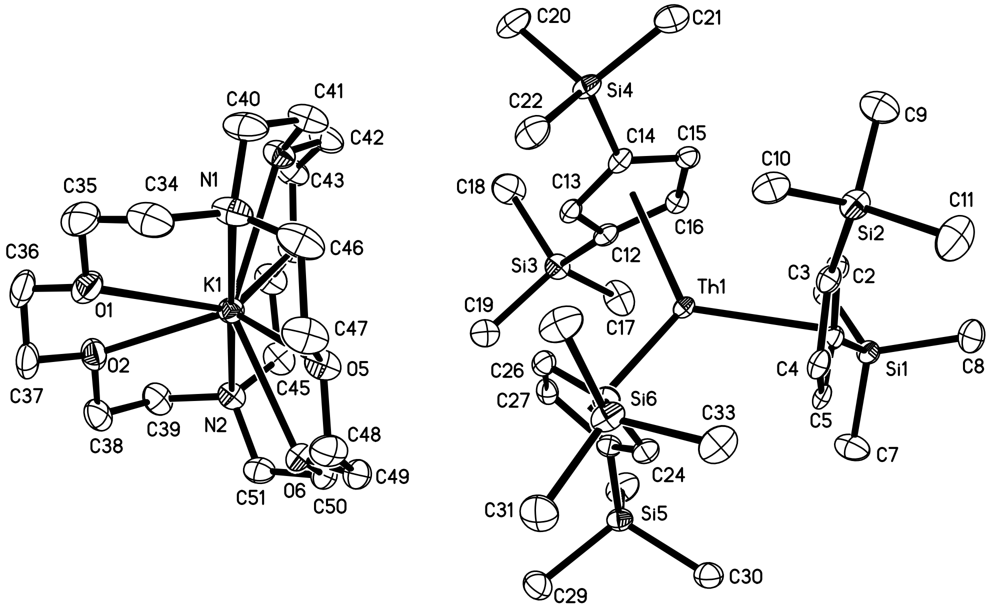

Figure S4, molecular structure of **2**, with thermal ellipsoids drawn at the 50% probability level and hydrogen atoms omitted for clarity.

Table S5. Crystal data and structure refinement for **2.**

Identification code	rrl19 (Ryan Langeslay)	
Empirical formula	C_{51} H_{99} K N_2 O_6 Si_6 Th	
Formula weight	1276.00	
Temperature	138(2) K	
Wavelength	0.71073 Å	
Crystal system	Triclinic	
Space group	$P\bar{1}$	
Unit cell dimensions	a = 12.1595(8) Å	□= 100.9296(8)°.
	b = 12.7622(8) Å	□= 104.4481(8)°.
	c = 22.2587(14) Å	□ = 95.5814(8)°.
Volume	3246.0(4) Å^3	
Z	2	
Density (calculated)	1.305 Mg/m^3	
Absorption coefficient	2.513 mm^{-1}	
F(000)	1320	

Crystal color	red
Crystal size	0.307 x 0.138 x 0.056 mm^3
Theta range for data collection	1.644 to 28.750°
Index ranges	$-16 \leq h \leq 16$, $-17 \leq k \leq 16$, $-29 \leq l \leq 28$
Reflections collected	38800
Independent reflections	15230 [R(int) = 0.0445]
Completeness to theta = 25.242°	99.8 %
Absorption correction	Numerical
Max. and min. transmission	0.9194 and 0.6059
Refinement method	Full-matrix least-squares on F^2
Data / restraints / parameters	15230 / 0 / 622
Goodness-of-fit on F^2	1.010
Final R indices [I>2sigma(I) = 13490 data]	R1 = 0.0287, wR2 = 0.0594
R indices (all data, 0.74 Å)	R1 = 0.0361, wR2 = 0.0621
Largest diff. peak and hole	0.835 and -0.634 e.Å^{-3}

Table S6. Bond lengths [Å] and angles [°] for **2.**

Cnt1-Th(1)	2.512
Cnt2-Th(1)	2.533
Cnt3-Th(1)	2.519
Th(1)-C(2)	2.739(3)
Th(1)-C(23)	2.744(3)
Th(1)-C(24)	2.750(3)
Th(1)-C(13)	2.755(3)
Th(1)-C(3)	2.770(3)
Th(1)-C(14)	2.776(3)
Th(1)-C(27)	2.780(3)
Th(1)-C(4)	2.800(3)
Th(1)-C(15)	2.806(3)
Th(1)-C(1)	2.806(3)
Th(1)-C(5)	2.822(2)
Th(1)-C(12)	2.846(3)
Th(1)-C(26)	2.849(3)
Th(1)-C(25)	2.851(3)
Th(1)-C(16)	2.854(2)

Si(1)-C(1)	1.846(3)
Si(1)-C(6)	1.862(3)
Si(1)-C(7)	1.863(3)
Si(1)-C(8)	1.875(3)
Si(2)-C(3)	1.842(3)
Si(2)-C(9)	1.867(3)
Si(2)-C(11)	1.871(3)
Si(2)-C(10)	1.871(3)
Si(3)-C(12)	1.846(3)
Si(3)-C(17)	1.861(3)
Si(3)-C(19)	1.867(3)
Si(3)-C(18)	1.879(3)
Si(4)-C(14)	1.840(3)
Si(4)-C(21)	1.865(3)
Si(4)-C(22)	1.867(3)
Si(4)-C(20)	1.881(3)
Si(5)-C(23)	1.846(3)
Si(5)-C(28)	1.868(3)
Si(5)-C(30)	1.873(3)
Si(5)-C(29)	1.880(3)
Si(6)-C(25)	1.847(3)
Si(6)-C(33)	1.869(3)
Si(6)-C(32)	1.874(3)
Si(6)-C(31)	1.876(3)
C(1)-C(2)	1.428(4)
C(1)-C(5)	1.433(4)
C(2)-C(3)	1.443(3)
C(3)-C(4)	1.432(4)
C(4)-C(5)	1.393(4)
C(12)-C(16)	1.422(4)
C(12)-C(13)	1.431(4)
C(13)-C(14)	1.439(4)
C(14)-C(15)	1.441(4)
C(15)-C(16)	1.398(4)
C(23)-C(27)	1.439(4)
C(23)-C(24)	1.440(3)

C(24)-C(25)	1.432(4)
C(25)-C(26)	1.430(4)
C(26)-C(27)	1.395(4)
K(1)-O(2)	2.806(2)
K(1)-O(1)	2.809(2)
K(1)-O(6)	2.821(2)
K(1)-O(5)	2.826(2)
K(1)-O(4)	2.841(2)
K(1)-O(3)	2.843(2)
K(1)-N(2)	3.045(2)
K(1)-N(1)	3.052(3)
O(1)-C(35)	1.418(4)
O(1)-C(36)	1.424(4)
O(2)-C(38)	1.420(4)
O(2)-C(37)	1.428(3)
O(3)-C(42)	1.418(3)
O(3)-C(41)	1.418(3)
O(4)-C(43)	1.418(3)
O(4)-C(44)	1.419(3)
O(5)-C(47)	1.414(4)
O(5)-C(48)	1.423(4)
O(6)-C(49)	1.417(3)
O(6)-C(50)	1.422(3)
N(1)-C(40)	1.466(4)
N(1)-C(46)	1.467(4)
N(1)-C(34)	1.472(4)
N(2)-C(39)	1.464(4)
N(2)-C(45)	1.470(4)
N(2)-C(51)	1.475(4)
C(34)-C(35)	1.485(5)
C(36)-C(37)	1.488(5)
C(38)-C(39)	1.499(4)
C(40)-C(41)	1.508(4)
C(42)-C(43)	1.496(4)
C(44)-C(45)	1.509(4)
C(46)-C(47)	1.508(5)

C(48)-C(49)	1.495(4)
C(50)-C(51)	1.501(4)
Cnt1-Th(1)-Cnt2	122.6
Cnt1-Th(1)-Cnt3	117.8
Cnt2-Th(1)-Cnt3	119.6
C(2)-Th(1)-C(23)	132.71(8)
C(2)-Th(1)-C(24)	118.00(8)
C(23)-Th(1)-C(24)	30.40(7)
C(2)-Th(1)-C(13)	121.86(8)
C(23)-Th(1)-C(13)	100.63(8)
C(24)-Th(1)-C(13)	120.05(8)
C(2)-Th(1)-C(3)	30.36(7)
C(23)-Th(1)-C(3)	125.99(8)
C(24)-Th(1)-C(3)	99.46(8)
C(13)-Th(1)-C(3)	131.71(8)
C(2)-Th(1)-C(14)	98.52(8)
C(23)-Th(1)-C(14)	128.11(8)
C(24)-Th(1)-C(14)	136.75(8)
C(13)-Th(1)-C(14)	30.17(8)
C(3)-Th(1)-C(14)	101.73(8)
C(2)-Th(1)-C(27)	162.89(8)
C(23)-Th(1)-C(27)	30.18(7)
C(24)-Th(1)-C(27)	48.44(8)
C(13)-Th(1)-C(27)	73.14(8)
C(3)-Th(1)-C(27)	145.99(8)
C(14)-Th(1)-C(27)	98.32(8)
C(2)-Th(1)-C(4)	48.35(8)
C(23)-Th(1)-C(4)	96.30(8)
C(24)-Th(1)-C(4)	71.74(8)
C(13)-Th(1)-C(4)	159.94(8)
C(3)-Th(1)-C(4)	29.78(8)
C(14)-Th(1)-C(4)	130.00(8)
C(27)-Th(1)-C(4)	120.16(8)
C(2)-Th(1)-C(15)	73.88(8)
C(23)-Th(1)-C(15)	145.25(8)

C(24)-Th(1)-C(15)	166.55(8)
C(13)-Th(1)-C(15)	48.09(8)
C(3)-Th(1)-C(15)	88.40(8)
C(14)-Th(1)-C(15)	29.92(7)
C(27)-Th(1)-C(15)	121.08(8)
C(4)-Th(1)-C(15)	117.64(8)
C(2)-Th(1)-C(1)	29.82(7)
C(23)-Th(1)-C(1)	105.00(8)
C(24)-Th(1)-C(1)	100.27(8)
C(13)-Th(1)-C(1)	134.37(8)
C(3)-Th(1)-C(1)	50.28(7)
C(14)-Th(1)-C(1)	122.24(8)
C(27)-Th(1)-C(1)	134.10(8)
C(4)-Th(1)-C(1)	48.75(8)
C(15)-Th(1)-C(1)	93.15(8)
C(2)-Th(1)-C(5)	48.03(8)
C(23)-Th(1)-C(5)	85.46(8)
C(24)-Th(1)-C(5)	72.43(8)
C(13)-Th(1)-C(5)	163.20(8)
C(3)-Th(1)-C(5)	48.86(8)
C(14)-Th(1)-C(5)	146.39(8)
C(27)-Th(1)-C(5)	115.28(8)
C(4)-Th(1)-C(5)	28.69(7)
C(15)-Th(1)-C(5)	120.60(8)
C(1)-Th(1)-C(5)	29.50(7)
C(2)-Th(1)-C(12)	109.16(8)
C(23)-Th(1)-C(12)	97.52(8)
C(24)-Th(1)-C(12)	126.38(8)
C(13)-Th(1)-C(12)	29.55(7)
C(3)-Th(1)-C(12)	133.83(8)
C(14)-Th(1)-C(12)	49.83(8)
C(27)-Th(1)-C(12)	79.78(8)
C(4)-Th(1)-C(12)	156.56(8)
C(15)-Th(1)-C(12)	48.06(8)
C(1)-Th(1)-C(12)	109.06(8)
C(5)-Th(1)-C(12)	134.59(8)

C(2)-Th(1)-C(26)	154.53(8)
C(23)-Th(1)-C(26)	49.04(8)
C(24)-Th(1)-C(26)	47.75(8)
C(13)-Th(1)-C(26)	75.27(8)
C(3)-Th(1)-C(26)	124.33(8)
C(14)-Th(1)-C(26)	89.42(8)
C(27)-Th(1)-C(26)	28.67(7)
C(4)-Th(1)-C(26)	108.86(8)
C(15)-Th(1)-C(26)	118.82(8)
C(1)-Th(1)-C(26)	148.02(8)
C(5)-Th(1)-C(26)	119.42(8)
C(12)-Th(1)-C(26)	94.49(8)
C(2)-Th(1)-C(25)	126.97(8)
C(23)-Th(1)-C(25)	50.15(8)
C(24)-Th(1)-C(25)	29.56(7)
C(13)-Th(1)-C(25)	102.97(8)
C(3)-Th(1)-C(25)	98.59(8)
C(14)-Th(1)-C(25)	109.54(8)
C(27)-Th(1)-C(25)	48.45(7)
C(4)-Th(1)-C(25)	79.81(8)
C(15)-Th(1)-C(25)	138.70(8)
C(1)-Th(1)-C(25)	122.46(8)
C(5)-Th(1)-C(25)	92.97(8)
C(12)-Th(1)-C(25)	123.45(8)
C(26)-Th(1)-C(25)	29.07(7)
C(2)-Th(1)-C(16)	80.64(8)
C(23)-Th(1)-C(16)	121.95(7)
C(24)-Th(1)-C(16)	152.35(8)
C(13)-Th(1)-C(16)	47.69(7)
C(3)-Th(1)-C(16)	105.36(8)
C(14)-Th(1)-C(16)	48.87(7)
C(27)-Th(1)-C(16)	108.54(7)
C(4)-Th(1)-C(16)	128.90(8)
C(15)-Th(1)-C(16)	28.59(7)
C(1)-Th(1)-C(16)	86.67(7)
C(5)-Th(1)-C(16)	115.90(7)

C(12)-Th(1)-C(16)	28.90(7)
C(26)-Th(1)-C(16)	121.57(7)
C(25)-Th(1)-C(16)	150.36(8)
C(1)-Si(1)-C(6)	114.42(13)
C(1)-Si(1)-C(7)	110.88(13)
C(6)-Si(1)-C(7)	108.60(15)
C(1)-Si(1)-C(8)	107.39(12)
C(6)-Si(1)-C(8)	107.57(14)
C(7)-Si(1)-C(8)	107.73(15)
C(3)-Si(2)-C(9)	107.39(13)
C(3)-Si(2)-C(11)	109.66(14)
C(9)-Si(2)-C(11)	107.06(16)
C(3)-Si(2)-C(10)	115.17(13)
C(9)-Si(2)-C(10)	110.95(14)
C(11)-Si(2)-C(10)	106.34(15)
C(12)-Si(3)-C(17)	111.66(13)
C(12)-Si(3)-C(19)	114.66(12)
C(17)-Si(3)-C(19)	111.27(14)
C(12)-Si(3)-C(18)	106.84(14)
C(17)-Si(3)-C(18)	108.55(16)
C(19)-Si(3)-C(18)	103.26(14)
C(14)-Si(4)-C(21)	110.94(13)
C(14)-Si(4)-C(22)	112.34(13)
C(21)-Si(4)-C(22)	112.19(14)
C(14)-Si(4)-C(20)	108.23(13)
C(21)-Si(4)-C(20)	106.50(15)
C(22)-Si(4)-C(20)	106.29(14)
C(23)-Si(5)-C(28)	117.53(13)
C(23)-Si(5)-C(30)	109.42(13)
C(28)-Si(5)-C(30)	106.99(14)
C(23)-Si(5)-C(29)	107.30(13)
C(28)-Si(5)-C(29)	106.98(14)
C(30)-Si(5)-C(29)	108.30(13)
C(25)-Si(6)-C(33)	112.81(13)
C(25)-Si(6)-C(32)	113.23(13)
C(33)-Si(6)-C(32)	109.96(15)

C(25)-Si(6)-C(31)	107.08(13)
C(33)-Si(6)-C(31)	106.01(14)
C(32)-Si(6)-C(31)	107.29(15)
C(2)-C(1)-C(5)	104.6(2)
C(2)-C(1)-Si(1)	126.3(2)
C(5)-C(1)-Si(1)	125.7(2)
C(2)-C(1)-Th(1)	72.47(14)
C(5)-C(1)-Th(1)	75.84(14)
Si(1)-C(1)-Th(1)	132.53(12)
C(1)-C(2)-C(3)	111.3(2)
C(1)-C(2)-Th(1)	77.72(15)
C(3)-C(2)-Th(1)	76.01(15)
C(4)-C(3)-C(2)	104.3(2)
C(4)-C(3)-Si(2)	127.38(19)
C(2)-C(3)-Si(2)	126.1(2)
C(4)-C(3)-Th(1)	76.29(15)
C(2)-C(3)-Th(1)	73.63(14)
Si(2)-C(3)-Th(1)	127.83(12)
C(5)-C(4)-C(3)	109.9(2)
C(5)-C(4)-Th(1)	76.51(15)
C(3)-C(4)-Th(1)	73.93(14)
C(4)-C(5)-C(1)	109.9(2)
C(4)-C(5)-Th(1)	74.80(15)
C(1)-C(5)-Th(1)	74.66(14)
C(16)-C(12)-C(13)	105.4(2)
C(16)-C(12)-Si(3)	125.87(19)
C(13)-C(12)-Si(3)	124.7(2)
C(16)-C(12)-Th(1)	75.87(14)
C(13)-C(12)-Th(1)	71.70(14)
Si(3)-C(12)-Th(1)	134.89(12)
C(12)-C(13)-C(14)	111.2(2)
C(12)-C(13)-Th(1)	78.75(15)
C(14)-C(13)-Th(1)	75.74(14)
C(13)-C(14)-C(15)	103.7(2)
C(13)-C(14)-Si(4)	124.6(2)
C(15)-C(14)-Si(4)	127.3(2)

C(13)-C(14)-Th(1)	74.09(14)
C(15)-C(14)-Th(1)	76.19(14)
Si(4)-C(14)-Th(1)	132.58(12)
C(16)-C(15)-C(14)	110.2(2)
C(16)-C(15)-Th(1)	77.60(15)
C(14)-C(15)-Th(1)	73.89(15)
C(15)-C(16)-C(12)	109.4(2)
C(15)-C(16)-Th(1)	73.81(14)
C(12)-C(16)-Th(1)	75.23(14)
C(27)-C(23)-C(24)	104.0(2)
C(27)-C(23)-Si(5)	129.2(2)
C(24)-C(23)-Si(5)	122.64(19)
C(27)-C(23)-Th(1)	76.30(14)
C(24)-C(23)-Th(1)	75.04(15)
Si(5)-C(23)-Th(1)	131.10(12)
C(25)-C(24)-C(23)	111.4(2)
C(25)-C(24)-Th(1)	79.12(15)
C(23)-C(24)-Th(1)	74.55(14)
C(26)-C(25)-C(24)	104.8(2)
C(26)-C(25)-Si(6)	126.3(2)
C(24)-C(25)-Si(6)	123.2(2)
C(26)-C(25)-Th(1)	75.40(14)
C(24)-C(25)-Th(1)	71.31(14)
Si(6)-C(25)-Th(1)	138.73(13)
C(27)-C(26)-C(25)	109.7(2)
C(27)-C(26)-Th(1)	72.91(15)
C(25)-C(26)-Th(1)	75.54(15)
C(26)-C(27)-C(23)	110.1(2)
C(26)-C(27)-Th(1)	78.42(15)
C(23)-C(27)-Th(1)	73.51(14)
O(2)-K(1)-O(1)	61.53(6)
O(2)-K(1)-O(6)	95.38(6)
O(1)-K(1)-O(6)	119.61(6)
O(2)-K(1)-O(5)	134.64(6)
O(1)-K(1)-O(5)	95.49(6)
O(6)-K(1)-O(5)	60.77(6)

O(2)-K(1)-O(4)	96.15(6)
O(1)-K(1)-O(4)	135.18(6)
O(6)-K(1)-O(4)	99.55(6)
O(5)-K(1)-O(4)	123.96(6)
O(2)-K(1)-O(3)	121.09(6)
O(1)-K(1)-O(3)	96.95(6)
O(6)-K(1)-O(3)	138.23(6)
O(5)-K(1)-O(3)	98.83(6)
O(4)-K(1)-O(3)	59.94(6)
O(2)-K(1)-N(2)	59.78(6)
O(1)-K(1)-N(2)	120.78(7)
O(6)-K(1)-N(2)	60.20(6)
O(5)-K(1)-N(2)	120.26(7)
O(4)-K(1)-N(2)	59.71(6)
O(3)-K(1)-N(2)	119.18(6)
O(2)-K(1)-N(1)	120.90(7)
O(1)-K(1)-N(1)	60.01(7)
O(6)-K(1)-N(1)	120.12(7)
O(5)-K(1)-N(1)	59.90(7)
O(4)-K(1)-N(1)	119.33(6)
O(3)-K(1)-N(1)	59.91(6)
N(2)-K(1)-N(1)	179.01(7)
C(35)-O(1)-C(36)	111.1(2)
C(35)-O(1)-K(1)	117.30(18)
C(36)-O(1)-K(1)	111.16(17)
C(38)-O(2)-C(37)	112.1(2)
C(38)-O(2)-K(1)	117.59(16)
C(37)-O(2)-K(1)	113.02(17)
C(42)-O(3)-C(41)	111.5(2)
C(42)-O(3)-K(1)	114.58(16)
C(41)-O(3)-K(1)	116.65(16)
C(43)-O(4)-C(44)	110.8(2)
C(43)-O(4)-K(1)	113.63(16)
C(44)-O(4)-K(1)	117.94(16)
C(47)-O(5)-C(48)	110.8(2)
C(47)-O(5)-K(1)	118.32(19)

C(48)-O(5)-K(1)	111.42(16)
C(49)-O(6)-C(50)	111.1(2)
C(49)-O(6)-K(1)	114.55(16)
C(50)-O(6)-K(1)	115.30(16)
C(40)-N(1)-C(46)	110.6(3)
C(40)-N(1)-C(34)	109.9(3)
C(46)-N(1)-C(34)	110.8(3)
C(40)-N(1)-K(1)	108.75(17)
C(46)-N(1)-K(1)	108.34(18)
C(34)-N(1)-K(1)	108.39(18)
C(39)-N(2)-C(45)	110.5(2)
C(39)-N(2)-C(51)	110.0(2)
C(45)-N(2)-C(51)	109.3(2)
C(39)-N(2)-K(1)	109.10(17)
C(45)-N(2)-K(1)	109.35(17)
C(51)-N(2)-K(1)	108.54(16)
N(1)-C(34)-C(35)	113.9(3)
O(1)-C(35)-C(34)	109.8(3)
O(1)-C(36)-C(37)	109.8(2)
O(2)-C(37)-C(36)	108.7(3)
O(2)-C(38)-C(39)	108.9(2)
N(2)-C(39)-C(38)	113.6(2)
N(1)-C(40)-C(41)	113.9(3)
O(3)-C(41)-C(40)	108.8(3)
O(3)-C(42)-C(43)	109.1(2)
O(4)-C(43)-C(42)	109.3(2)
O(4)-C(44)-C(45)	109.1(2)
N(2)-C(45)-C(44)	113.5(3)
N(1)-C(46)-C(47)	113.7(3)
O(5)-C(47)-C(46)	109.3(3)
O(5)-C(48)-C(49)	109.4(2)
O(6)-C(49)-C(48)	109.0(2)
O(6)-C(50)-C(51)	108.4(2)
N(2)-C(51)-C(50)	113.8(2)

X-ray Data Collection, Structure Solution and Refinement for 3.

A red crystal of approximate dimensions 0.262 x 0.255 x 0.254 mm was mounted in a cryo loop and transferred to a Bruker SMART APEX II diffractometer. The APEX2[26] program package was used to determine the unit-cell parameters and for data collection (20 sec/frame scan time for a sphere of diffraction data). The raw frame data was processed using SAINT[21] and SADABS[22] to yield the reflection data file. Subsequent calculations were carried out using the SHELXTL[23] program. The diffraction symmetry was 2/*m* and the systematic absences were consistent with the monoclinic space group $P2_1/n$ that was later determined to be correct.

The structure was solved by direct methods and refined on F^2 by full-matrix least-squares techniques. The analytical scattering factors[25] for neutral atoms were used throughout the analysis. C(33) and C(52) were disordered and included using multiple components with partial site-occupancy factors. Hydrogen atoms were included using a riding model.

At convergence, wR2 = 0.0976 and Goof = 1.100 for 632 variables refined against 14108 data (0.80 Å), R1 = 0.0384 for those 11825 data with I > 2.0σ(I).

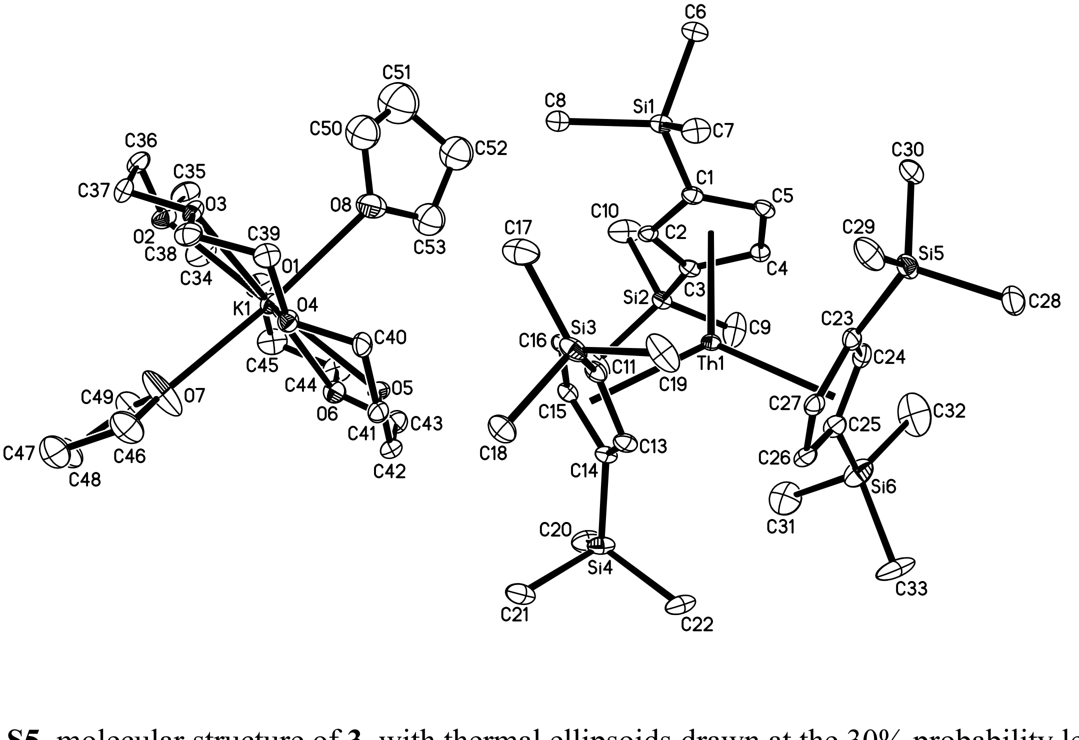

Figure S5, molecular structure of **3**, with thermal ellipsoids drawn at the 30% probability level and hydrogen atoms omitted for clarity.

Table S7. Crystal data and structure refinement for **3.**

Identification code	rrl20 (Ryan Langeslay)	
Empirical formula	$C_{53} H_{103} K O_8 Si_6 Th$	
Formula weight	1308.03	
Temperature	133(2) K	
Wavelength	0.71073 Å	
Crystal system	Monoclinic	
Space group	$P2_1/n$	
Unit cell dimensions	a = 17.1884(16) Å	α= 90°.
	b = 18.0161(16) Å	β= 99.6558(11)°.
	c = 22.654(2) Å	γ = 90°.
Volume	6915.8(11) $Å^3$	
Z	4	
Density (calculated)	1.256 Mg/m^3	
Absorption coefficient	2.362 mm^{-1}	
F(000)	2712	
Crystal color	red	
Crystal size	0.262 x 0.255 x 0.254 mm^3	
Theta range for data collection	1.381 to 26.370°	
Index ranges	$-21 \le h \le 21$, $-22 \le k \le 22$, $-28 \le l \le 28$	
Reflections collected	73786	
Independent reflections	14108 [R(int) = 0.0441]	
Completeness to theta = 25.242°	99.9 %	
Absorption correction	Numerical	
Max. and min. transmission	0.7098 and 0.6224	
Refinement method	Full-matrix least-squares on F^2	
Data / restraints / parameters	14108 / 0 / 632	
Goodness-of-fit on F^2	1.100	
Final R indices [I>2sigma(I) = 11825 data]	R1 = 0.0384, wR2 = 0.0921	
R indices (all data, 0.8 Å)	R1 = 0.0511, wR2 = 0.0976	
Largest diff. peak and hole	2.171 and -0.878 e.$Å^{-3}$	

Table S8. Bond lengths [Å] and angles [°] for **3**.

Cnt1-Th(1)	2.529
Cnt2-Th(1)	2.522
Cnt3-Th(1)	2.524
Th(1)-C(13)	2.732(4)
Th(1)-C(2)	2.749(4)
Th(1)-C(24)	2.756(4)
Th(1)-C(12)	2.770(4)
Th(1)-C(25)	2.771(5)
Th(1)-C(1)	2.779(4)
Th(1)-C(26)	2.796(4)
Th(1)-C(16)	2.806(4)
Th(1)-C(3)	2.822(4)
Th(1)-C(14)	2.829(4)
Th(1)-C(27)	2.829(4)
Th(1)-C(5)	2.832(4)
Th(1)-C(15)	2.841(4)
Th(1)-C(4)	2.844(4)
Th(1)-C(23)	2.846(4)
Si(1)-C(1)	1.849(5)
Si(1)-C(7)	1.867(5)
Si(1)-C(8)	1.871(5)
Si(1)-C(6)	1.888(5)
Si(2)-C(3)	1.855(4)
Si(2)-C(9)	1.856(6)
Si(2)-C(11)	1.861(6)
Si(2)-C(10)	1.881(6)
Si(3)-C(12)	1.850(5)
Si(3)-C(19)	1.854(6)
Si(3)-C(17)	1.873(6)
Si(3)-C(18)	1.882(5)
Si(4)-C(14)	1.849(5)
Si(4)-C(20)	1.859(6)
Si(4)-C(22)	1.864(5)
Si(4)-C(21)	1.884(6)
Si(5)-C(23)	1.857(5)

Si(5)-C(30)	1.866(5)
Si(5)-C(29)	1.869(6)
Si(5)-C(28)	1.878(6)
Si(6)-C(33A)	1.81(3)
Si(6)-C(32)	1.851(7)
Si(6)-C(25)	1.857(5)
Si(6)-C(31)	1.869(7)
Si(6)-C(33)	1.97(2)
C(1)-C(5)	1.438(6)
C(1)-C(2)	1.447(6)
C(2)-C(3)	1.432(6)
C(3)-C(4)	1.426(6)
C(4)-C(5)	1.397(6)
C(12)-C(16)	1.427(6)
C(12)-C(13)	1.435(6)
C(13)-C(14)	1.432(6)
C(14)-C(15)	1.412(6)
C(15)-C(16)	1.389(6)
C(23)-C(24)	1.426(7)
C(23)-C(27)	1.428(6)
C(24)-C(25)	1.434(7)
C(25)-C(26)	1.436(7)
C(26)-C(27)	1.403(7)
K(1)-O(7)	2.659(4)
K(1)-O(8)	2.697(5)
K(1)-O(5)	2.762(3)
K(1)-O(4)	2.773(3)
K(1)-O(3)	2.791(3)
K(1)-O(1)	2.814(4)
K(1)-O(2)	2.818(3)
K(1)-O(6)	2.823(3)
O(1)-C(34)	1.424(7)
O(1)-C(45)	1.424(7)
O(2)-C(35)	1.422(6)
O(2)-C(36)	1.432(6)
O(3)-C(38)	1.422(6)

O(3)-C(37)	1.431(6)
O(4)-C(40)	1.416(5)
O(4)-C(39)	1.426(6)
O(5)-C(42)	1.425(6)
O(5)-C(41)	1.425(6)
O(6)-C(44)	1.420(6)
O(6)-C(43)	1.423(6)
O(7)-C(46)	1.357(7)
O(7)-C(49)	1.396(7)
O(8)-C(53)	1.389(8)
O(8)-C(50)	1.406(9)
C(34)-C(35)	1.491(9)
C(36)-C(37)	1.493(8)
C(38)-C(39)	1.491(7)
C(40)-C(41)	1.494(7)
C(42)-C(43)	1.500(8)
C(44)-C(45)	1.499(8)
C(46)-C(47)	1.477(9)
C(47)-C(48)	1.516(8)
C(48)-C(49)	1.501(8)
C(50)-C(51)	1.455(12)
C(51)-C(52)	1.498(18)
C(51)-C(52A)	1.62(2)
C(52)-C(53)	1.451(17)
C(52A)-C(53)	1.649(19)

Cnt1-Th(1)-Cnt2	120.5
Cnt1-Th(1)-Cnt3	119.4
Cnt2-Th(1)-Cnt3	120.1
C(13)-Th(1)-C(2)	120.29(13)
C(13)-Th(1)-C(24)	119.64(14)
C(2)-Th(1)-C(24)	120.07(13)
C(13)-Th(1)-C(12)	30.22(13)
C(2)-Th(1)-C(12)	98.67(14)
C(24)-Th(1)-C(12)	135.14(14)
C(13)-Th(1)-C(25)	98.79(14)

C(2)-Th(1)-C(25)	135.47(14)
C(24)-Th(1)-C(25)	30.07(14)
C(12)-Th(1)-C(25)	125.17(14)
C(13)-Th(1)-C(1)	130.61(14)
C(2)-Th(1)-C(1)	30.34(12)
C(24)-Th(1)-C(1)	102.13(14)
C(12)-Th(1)-C(1)	101.03(13)
C(25)-Th(1)-C(1)	129.14(14)
C(13)-Th(1)-C(26)	72.33(14)
C(2)-Th(1)-C(26)	165.36(14)
C(24)-Th(1)-C(26)	48.11(14)
C(12)-Th(1)-C(26)	95.76(14)
C(25)-Th(1)-C(26)	29.89(14)
C(1)-Th(1)-C(26)	147.25(14)
C(13)-Th(1)-C(16)	48.11(13)
C(2)-Th(1)-C(16)	72.79(13)
C(24)-Th(1)-C(16)	164.79(14)
C(12)-Th(1)-C(16)	29.65(13)
C(25)-Th(1)-C(16)	145.26(14)
C(1)-Th(1)-C(16)	85.57(13)
C(26)-Th(1)-C(16)	120.41(13)
C(13)-Th(1)-C(3)	133.04(13)
C(2)-Th(1)-C(3)	29.75(13)
C(24)-Th(1)-C(3)	101.28(13)
C(12)-Th(1)-C(3)	122.97(13)
C(25)-Th(1)-C(3)	107.38(14)
C(1)-Th(1)-C(3)	50.12(13)
C(26)-Th(1)-C(3)	136.43(13)
C(16)-Th(1)-C(3)	93.73(13)
C(13)-Th(1)-C(14)	29.79(13)
C(2)-Th(1)-C(14)	105.15(13)
C(24)-Th(1)-C(14)	127.81(14)
C(12)-Th(1)-C(14)	49.81(13)
C(25)-Th(1)-C(14)	98.64(14)
C(1)-Th(1)-C(14)	129.59(13)
C(26)-Th(1)-C(14)	82.41(14)

C(16)-Th(1)-C(14)	48.01(13)
C(3)-Th(1)-C(14)	106.49(13)
C(13)-Th(1)-C(27)	76.31(13)
C(2)-Th(1)-C(27)	154.09(13)
C(24)-Th(1)-C(27)	47.69(14)
C(12)-Th(1)-C(27)	87.59(14)
C(25)-Th(1)-C(27)	48.95(14)
C(1)-Th(1)-C(27)	123.82(13)
C(26)-Th(1)-C(27)	28.88(13)
C(16)-Th(1)-C(27)	117.16(13)
C(3)-Th(1)-C(27)	148.80(13)
C(14)-Th(1)-C(27)	98.11(13)
C(13)-Th(1)-C(5)	159.20(14)
C(2)-Th(1)-C(5)	48.18(13)
C(24)-Th(1)-C(5)	74.36(13)
C(12)-Th(1)-C(5)	128.98(13)
C(25)-Th(1)-C(5)	99.58(14)
C(1)-Th(1)-C(5)	29.68(13)
C(26)-Th(1)-C(5)	122.29(14)
C(16)-Th(1)-C(5)	114.97(13)
C(3)-Th(1)-C(5)	48.42(12)
C(14)-Th(1)-C(5)	152.76(13)
C(27)-Th(1)-C(5)	109.13(13)
C(13)-Th(1)-C(15)	47.76(13)
C(2)-Th(1)-C(15)	77.10(13)
C(24)-Th(1)-C(15)	154.59(14)
C(12)-Th(1)-C(15)	48.55(13)
C(25)-Th(1)-C(15)	124.52(14)
C(1)-Th(1)-C(15)	100.90(13)
C(26)-Th(1)-C(15)	111.25(14)
C(16)-Th(1)-C(15)	28.46(13)
C(3)-Th(1)-C(15)	85.42(13)
C(14)-Th(1)-C(15)	28.84(13)
C(27)-Th(1)-C(15)	123.72(13)
C(5)-Th(1)-C(15)	125.28(13)
C(13)-Th(1)-C(4)	161.75(13)

C(2)-Th(1)-C(4)	47.70(13)
C(24)-Th(1)-C(4)	74.26(13)
C(12)-Th(1)-C(4)	146.03(13)
C(25)-Th(1)-C(4)	88.79(13)
C(1)-Th(1)-C(4)	48.58(13)
C(26)-Th(1)-C(4)	118.09(13)
C(16)-Th(1)-C(4)	119.89(13)
C(3)-Th(1)-C(4)	29.15(12)
C(14)-Th(1)-C(4)	132.81(13)
C(27)-Th(1)-C(4)	120.38(13)
C(5)-Th(1)-C(4)	28.50(13)
C(15)-Th(1)-C(4)	114.53(13)
C(13)-Th(1)-C(23)	104.67(13)
C(2)-Th(1)-C(23)	127.36(13)
C(24)-Th(1)-C(23)	29.43(13)
C(12)-Th(1)-C(23)	108.94(13)
C(25)-Th(1)-C(23)	49.85(14)
C(1)-Th(1)-C(23)	99.19(13)
C(26)-Th(1)-C(23)	48.45(14)
C(16)-Th(1)-C(23)	136.98(13)
C(3)-Th(1)-C(23)	122.08(13)
C(14)-Th(1)-C(23)	127.02(13)
C(27)-Th(1)-C(23)	29.15(13)
C(5)-Th(1)-C(23)	80.19(13)
C(15)-Th(1)-C(23)	152.43(13)
C(4)-Th(1)-C(23)	92.94(13)
C(1)-Si(1)-C(7)	116.2(2)
C(1)-Si(1)-C(8)	108.8(2)
C(7)-Si(1)-C(8)	110.0(2)
C(1)-Si(1)-C(6)	109.2(2)
C(7)-Si(1)-C(6)	105.5(2)
C(8)-Si(1)-C(6)	106.7(2)
C(3)-Si(2)-C(9)	109.6(2)
C(3)-Si(2)-C(11)	114.8(2)
C(9)-Si(2)-C(11)	111.4(3)
C(3)-Si(2)-C(10)	109.3(2)

C(9)-Si(2)-C(10)	107.8(3)
C(11)-Si(2)-C(10)	103.6(3)
C(12)-Si(3)-C(19)	113.5(2)
C(12)-Si(3)-C(17)	112.1(2)
C(19)-Si(3)-C(17)	110.2(3)
C(12)-Si(3)-C(18)	107.1(2)
C(19)-Si(3)-C(18)	106.4(2)
C(17)-Si(3)-C(18)	107.1(3)
C(14)-Si(4)-C(20)	113.4(2)
C(14)-Si(4)-C(22)	113.0(2)
C(20)-Si(4)-C(22)	110.7(3)
C(14)-Si(4)-C(21)	107.1(2)
C(20)-Si(4)-C(21)	107.4(3)
C(22)-Si(4)-C(21)	104.6(3)
C(23)-Si(5)-C(30)	114.3(2)
C(23)-Si(5)-C(29)	111.6(2)
C(30)-Si(5)-C(29)	109.5(3)
C(23)-Si(5)-C(28)	107.8(3)
C(30)-Si(5)-C(28)	105.8(2)
C(29)-Si(5)-C(28)	107.5(3)
C(33A)-Si(6)-C(32)	101.5(9)
C(33A)-Si(6)-C(25)	107.1(8)
C(32)-Si(6)-C(25)	107.8(3)
C(33A)-Si(6)-C(31)	114.6(9)
C(32)-Si(6)-C(31)	107.1(3)
C(25)-Si(6)-C(31)	117.5(3)
C(32)-Si(6)-C(33)	118.7(9)
C(25)-Si(6)-C(33)	107.2(8)
C(31)-Si(6)-C(33)	98.9(7)
C(5)-C(1)-C(2)	104.4(4)
C(5)-C(1)-Si(1)	127.9(3)
C(2)-C(1)-Si(1)	126.0(3)
C(5)-C(1)-Th(1)	77.2(2)
C(2)-C(1)-Th(1)	73.7(2)
Si(1)-C(1)-Th(1)	125.4(2)
C(3)-C(2)-C(1)	111.0(4)

C(3)-C(2)-Th(1)	78.0(2)
C(1)-C(2)-Th(1)	76.0(2)
C(4)-C(3)-C(2)	104.7(4)
C(4)-C(3)-Si(2)	124.8(3)
C(2)-C(3)-Si(2)	127.9(3)
C(4)-C(3)-Th(1)	76.3(2)
C(2)-C(3)-Th(1)	72.3(2)
Si(2)-C(3)-Th(1)	130.3(2)
C(5)-C(4)-C(3)	110.4(4)
C(5)-C(4)-Th(1)	75.3(2)
C(3)-C(4)-Th(1)	74.6(2)
C(4)-C(5)-C(1)	109.4(4)
C(4)-C(5)-Th(1)	76.2(2)
C(1)-C(5)-Th(1)	73.1(2)
C(16)-C(12)-C(13)	104.2(4)
C(16)-C(12)-Si(3)	128.0(3)
C(13)-C(12)-Si(3)	122.4(3)
C(16)-C(12)-Th(1)	76.5(2)
C(13)-C(12)-Th(1)	73.4(2)
Si(3)-C(12)-Th(1)	135.0(2)
C(14)-C(13)-C(12)	110.7(4)
C(14)-C(13)-Th(1)	78.8(2)
C(12)-C(13)-Th(1)	76.4(2)
C(15)-C(14)-C(13)	105.1(4)
C(15)-C(14)-Si(4)	128.2(3)
C(13)-C(14)-Si(4)	121.8(3)
C(15)-C(14)-Th(1)	76.1(2)
C(13)-C(14)-Th(1)	71.4(2)
Si(4)-C(14)-Th(1)	136.5(2)
C(16)-C(15)-C(14)	109.9(4)
C(16)-C(15)-Th(1)	74.4(3)
C(14)-C(15)-Th(1)	75.1(3)
C(15)-C(16)-C(12)	110.1(4)
C(15)-C(16)-Th(1)	77.2(3)
C(12)-C(16)-Th(1)	73.8(2)
C(24)-C(23)-C(27)	104.6(4)

C(24)-C(23)-Si(5)	126.3(3)
C(27)-C(23)-Si(5)	125.4(3)
C(24)-C(23)-Th(1)	71.8(2)
C(27)-C(23)-Th(1)	74.7(2)
Si(5)-C(23)-Th(1)	134.9(2)
C(23)-C(24)-C(25)	111.8(4)
C(23)-C(24)-Th(1)	78.8(3)
C(25)-C(24)-Th(1)	75.5(3)
C(24)-C(25)-C(26)	104.1(4)
C(24)-C(25)-Si(6)	124.9(4)
C(26)-C(25)-Si(6)	128.5(4)
C(24)-C(25)-Th(1)	74.4(3)
C(26)-C(25)-Th(1)	76.0(3)
Si(6)-C(25)-Th(1)	127.9(2)
C(27)-C(26)-C(25)	109.6(4)
C(27)-C(26)-Th(1)	76.9(3)
C(25)-C(26)-Th(1)	74.1(3)
C(26)-C(27)-C(23)	109.7(4)
C(26)-C(27)-Th(1)	74.3(3)
C(23)-C(27)-Th(1)	76.1(2)
O(7)-K(1)-O(8)	164.38(16)
O(7)-K(1)-O(5)	98.08(16)
O(8)-K(1)-O(5)	80.94(13)
O(7)-K(1)-O(4)	83.70(12)
O(8)-K(1)-O(4)	82.12(14)
O(5)-K(1)-O(4)	61.94(9)
O(7)-K(1)-O(3)	85.61(16)
O(8)-K(1)-O(3)	81.80(12)
O(5)-K(1)-O(3)	121.82(10)
O(4)-K(1)-O(3)	60.86(9)
O(7)-K(1)-O(1)	98.63(13)
O(8)-K(1)-O(1)	95.62(15)
O(5)-K(1)-O(1)	117.05(11)
O(4)-K(1)-O(1)	177.61(11)
O(3)-K(1)-O(1)	119.67(11)
O(7)-K(1)-O(2)	82.44(16)

O(8)-K(1)-O(2)	99.10(14)
O(5)-K(1)-O(2)	177.91(11)
O(4)-K(1)-O(2)	120.15(10)
O(3)-K(1)-O(2)	60.21(10)
O(1)-K(1)-O(2)	60.86(11)
O(7)-K(1)-O(6)	91.74(16)
O(8)-K(1)-O(6)	101.07(12)
O(5)-K(1)-O(6)	60.12(10)
O(4)-K(1)-O(6)	120.48(10)
O(3)-K(1)-O(6)	176.90(10)
O(1)-K(1)-O(6)	59.12(11)
O(2)-K(1)-O(6)	117.88(11)
C(34)-O(1)-C(45)	112.3(4)
C(34)-O(1)-K(1)	115.1(3)
C(45)-O(1)-K(1)	115.5(3)
C(35)-O(2)-C(36)	112.1(4)
C(35)-O(2)-K(1)	110.2(3)
C(36)-O(2)-K(1)	114.9(3)
C(38)-O(3)-C(37)	112.6(4)
C(38)-O(3)-K(1)	114.3(3)
C(37)-O(3)-K(1)	114.2(3)
C(40)-O(4)-C(39)	112.1(3)
C(40)-O(4)-K(1)	111.0(3)
C(39)-O(4)-K(1)	113.1(3)
C(42)-O(5)-C(41)	111.9(4)
C(42)-O(5)-K(1)	115.9(3)
C(41)-O(5)-K(1)	114.3(3)
C(44)-O(6)-C(43)	111.9(4)
C(44)-O(6)-K(1)	117.2(3)
C(43)-O(6)-K(1)	115.5(3)
C(46)-O(7)-C(49)	111.9(5)
C(46)-O(7)-K(1)	135.1(4)
C(49)-O(7)-K(1)	112.8(3)
C(53)-O(8)-C(50)	109.4(6)
C(53)-O(8)-K(1)	113.9(4)
C(50)-O(8)-K(1)	127.7(4)

O(1)-C(34)-C(35)	109.2(5)
O(2)-C(35)-C(34)	109.2(5)
O(2)-C(36)-C(37)	108.9(4)
O(3)-C(37)-C(36)	107.6(4)
O(3)-C(38)-C(39)	107.9(4)
O(4)-C(39)-C(38)	108.5(4)
O(4)-C(40)-C(41)	109.5(4)
O(5)-C(41)-C(40)	108.8(4)
O(5)-C(42)-C(43)	108.6(4)
O(6)-C(43)-C(42)	109.1(4)
O(6)-C(44)-C(45)	108.4(4)
O(1)-C(45)-C(44)	108.4(5)
O(7)-C(46)-C(47)	108.5(5)
C(46)-C(47)-C(48)	103.8(5)
C(49)-C(48)-C(47)	103.9(5)
O(7)-C(49)-C(48)	107.3(5)
O(8)-C(50)-C(51)	106.2(7)
C(50)-C(51)-C(52)	100.4(10)
C(50)-C(51)-C(52A)	107.6(10)
C(53)-C(52)-C(51)	104.9(12)
C(51)-C(52A)-C(53)	91.3(11)
O(8)-C(53)-C(52)	106.5(9)
O(8)-C(53)-C(52A)	101.6(8)

X-ray Data Collection, Structure Solution and Refinement for 4.

A yellow crystal of approximate dimensions 0.168 x 0.217 x 0.300 mm was mounted on a glass fiber and transferred to a Bruker SMART APEX II diffractometer. The APEX2[20] program package was used to determine the unit-cell parameters and for data collection (20 sec/frame scan time for a sphere of diffraction data). The raw frame data was processed using SAINT[21] and SADABS[22] to yield the reflection data file. Subsequent calculations were carried out using the SHELXTL[23] program. The diffraction symmetry was 2/*m* and the systematic absences were consistent with the monoclinic space groups *Cc* and *C*2/*c*. It was later determined that space group *C*2/*c* was correct.

The structure was solved by direct methods and refined on F^2 by full-matrix least-squares techniques. The analytical scattering factors[25] for neutral atoms were used throughout the

analysis. Hydrogen atoms were included using a riding model. The molecule was located on a two-fold rotation axis.

At convergence, wR2 = 0.0586 and Goof = 1.399 for 165 variables refined against 3882 data (0.74Å), R1 = 0.0261 for those 3882 data with I > 2.0σ(I).

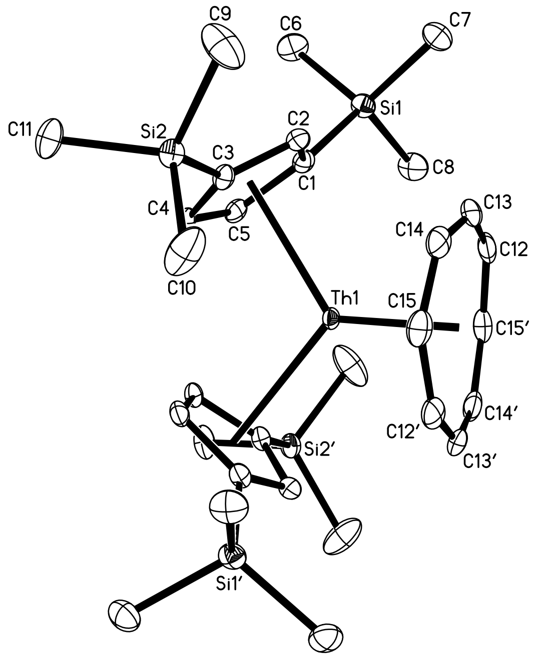

Figure S6, molecular structure of **4**, with thermal ellipsoids drawn at the 50% probability level and hydrogen atoms omitted for clarity.

Table S9. Crystal data and structure refinement for **4**.

Identification code	rrl32 (Ryan Langeslay)
Empirical formula	$C_{30} H_{50} Si_4 Th$
Formula weight	755.10
Temperature	133(2) K
Wavelength	0.71073 Å
Crystal system	Monoclinic

Space group	*C*2/*c*	
Unit cell dimensions	a = 20.2007(16) Å	α= 90°.
	b = 8.1694(6) Å	β= 91.3531(9)°.
	c = 19.9544(15) Å	γ = 90°.
Volume	3292.1(4) Å^3	
Z	4	
Density (calculated)	1.523 Mg/m^3	
Absorption coefficient	4.692 mm^{-1}	
F(000)	1504	
Crystal color	yellow	
Crystal size	0.300 x 0.217 x 0.168 mm^3	
Theta range for data collection	2.017 to 28.641°	
Index ranges	$-27 \leq h \leq 26$, $-10 \leq k \leq 10$, $-25 \leq l \leq 26$	
Reflections collected	17433	
Independent reflections	3959 [R(int) = 0.0187]	
Completeness to theta = 25.242°	100.0 %	
Absorption correction	Numerical	
Max. and min. transmission	0.6061 and 0.3757	
Refinement method	Full-matrix least-squares on F^2	
Data / restraints / parameters	3959 / 0 / 165	
Goodness-of-fit on F^2	1.399	
Final R indices [I>2sigma(I) = 3882 data]	R1 = 0.0261, wR2 = 0.0584	
R indices (all data, 0.74Å)	R1 = 0.0270, wR2 = 0.0586	
Largest diff. peak and hole	1.912 and -3.390 e.Å^{-3}	

Table S10. Bond lengths [Å] and angles [°] for **4**.

Th(1)-Cnt1	2.611
Th(1)-Cnt2	2.106
Th(1)-C(15)	2.736(4)
Th(1)-C(14)	2.769(3)
Th(1)-C(12)	2.815(4)
Th(1)-C(13)	2.841(4)
Th(1)-C(4)	2.852(3)

Th(1)-C(5)	2.853(3)
Th(1)-C(3)	2.872(3)
Th(1)-C(2)	2.894(3)
Th(1)-C(1)	2.915(3)
Si(1)-C(6)	1.870(4)
Si(1)-C(7)	1.872(4)
Si(1)-C(1)	1.873(3)
Si(1)-C(8)	1.874(4)
Si(2)-C(10)	1.861(4)
Si(2)-C(9)	1.862(4)
Si(2)-C(11)	1.864(4)
Si(2)-C(3)	1.870(3)
C(1)-C(5)	1.425(5)
C(1)-C(2)	1.425(5)
C(2)-C(3)	1.426(5)
C(3)-C(4)	1.421(5)
C(4)-C(5)	1.405(5)
C(12)-C(13)	1.403(6)
C(12)-C(15)#1	1.403(5)
C(13)-C(14)	1.399(6)
C(14)-C(15)	1.409(5)

Cnt1-Th(1)-Cnt1'	107.9
Cnt1-Th(1)-Cnt2	126.0
Cnt1-Th(1)-C(12)	94.4
Cnt1-Th(1)-C(13)	86.8
Cnt1-Th(1)-C(14)	93.7
Cnt1-Th(1)-C(15)	116.0
Cnt1-Th(1)-C(12')	145.1
Cnt1-Th(1)-C(13')	165.3
Cnt1-Th(1)-C(14')	144.5
Cnt1-Th(1)-C(15')	115.2
C(15)-Th(1)-C(15)#1	85.39(16)
C(15)-Th(1)-C(14)	29.65(11)
C(15)#1-Th(1)-C(14)	76.78(11)
C(15)-Th(1)-C(14)#1	76.78(11)

C(15)#1-Th(1)-C(14)#1	29.65(11)
C(14)-Th(1)-C(14)#1	83.37(16)
C(15)-Th(1)-C(12)	75.62(11)
C(15)#1-Th(1)-C(12)	29.25(11)
C(14)-Th(1)-C(12)	55.59(12)
C(14)#1-Th(1)-C(12)	54.98(11)
C(15)-Th(1)-C(12)#1	29.24(11)
C(15)#1-Th(1)-C(12)#1	75.62(11)
C(14)-Th(1)-C(12)#1	54.98(11)
C(14)#1-Th(1)-C(12)#1	55.59(12)
C(12)-Th(1)-C(12)#1	80.62(15)
C(15)-Th(1)-C(13)#1	55.43(11)
C(15)#1-Th(1)-C(13)#1	55.18(11)
C(14)-Th(1)-C(13)#1	73.69(11)
C(14)#1-Th(1)-C(13)#1	28.84(12)
C(12)-Th(1)-C(13)#1	72.29(11)
C(12)#1-Th(1)-C(13)#1	28.72(11)
C(15)-Th(1)-C(13)	55.18(11)
C(15)#1-Th(1)-C(13)	55.43(11)
C(14)-Th(1)-C(13)	28.84(12)
C(14)#1-Th(1)-C(13)	73.69(11)
C(12)-Th(1)-C(13)	28.72(11)
C(12)#1-Th(1)-C(13)	72.29(11)
C(13)#1-Th(1)-C(13)	78.49(15)
C(15)-Th(1)-C(4)#1	135.90(11)
C(15)#1-Th(1)-C(4)#1	122.48(11)
C(14)-Th(1)-C(4)#1	159.45(11)
C(14)#1-Th(1)-C(4)#1	109.82(10)
C(12)-Th(1)-C(4)#1	144.92(10)
C(12)#1-Th(1)-C(4)#1	118.94(10)
C(13)#1-Th(1)-C(4)#1	110.04(10)
C(13)-Th(1)-C(4)#1	168.49(10)
C(15)-Th(1)-C(4)	122.48(11)
C(15)#1-Th(1)-C(4)	135.90(11)
C(14)-Th(1)-C(4)	109.82(10)
C(14)#1-Th(1)-C(4)	159.45(11)

C(12)-Th(1)-C(4)	118.94(10)
C(12)#1-Th(1)-C(4)	144.92(10)
C(13)#1-Th(1)-C(4)	168.49(10)
C(13)-Th(1)-C(4)	110.04(10)
C(4)#1-Th(1)-C(4)	62.73(13)
C(15)-Th(1)-C(5)#1	108.30(11)
C(15)#1-Th(1)-C(5)#1	140.79(11)
C(14)-Th(1)-C(5)#1	131.62(11)
C(14)#1-Th(1)-C(5)#1	116.25(10)
C(12)-Th(1)-C(5)#1	169.97(10)
C(12)#1-Th(1)-C(5)#1	98.34(10)
C(13)#1-Th(1)-C(5)#1	102.01(10)
C(13)-Th(1)-C(5)#1	159.95(11)
C(4)#1-Th(1)-C(5)#1	28.52(9)
C(4)-Th(1)-C(5)#1	67.27(10)
C(15)-Th(1)-C(5)	140.79(11)
C(15)#1-Th(1)-C(5)	108.30(11)
C(14)-Th(1)-C(5)	116.25(10)
C(14)#1-Th(1)-C(5)	131.62(11)
C(12)-Th(1)-C(5)	98.34(10)
C(12)#1-Th(1)-C(5)	169.97(10)
C(13)#1-Th(1)-C(5)	159.95(11)
C(13)-Th(1)-C(5)	102.01(10)
C(4)#1-Th(1)-C(5)	67.27(10)
C(4)-Th(1)-C(5)	28.52(9)
C(5)#1-Th(1)-C(5)	84.41(14)
C(15)-Th(1)-C(3)	95.82(11)
C(15)#1-Th(1)-C(3)	132.09(11)
C(14)-Th(1)-C(3)	81.21(10)
C(14)#1-Th(1)-C(3)	159.54(10)
C(12)-Th(1)-C(3)	104.87(10)
C(12)#1-Th(1)-C(3)	122.95(11)
C(13)#1-Th(1)-C(3)	151.18(10)
C(13)-Th(1)-C(3)	86.38(10)
C(4)#1-Th(1)-C(3)	88.88(10)
C(4)-Th(1)-C(3)	28.74(9)

C(5)#1-Th(1)-C(3)	84.13(10)
C(5)-Th(1)-C(3)	47.51(9)
C(15)-Th(1)-C(3)#1	132.09(11)
C(15)#1-Th(1)-C(3)#1	95.82(11)
C(14)-Th(1)-C(3)#1	159.54(10)
C(14)#1-Th(1)-C(3)#1	81.21(10)
C(12)-Th(1)-C(3)#1	122.95(11)
C(12)#1-Th(1)-C(3)#1	104.87(10)
C(13)#1-Th(1)-C(3)#1	86.38(10)
C(13)-Th(1)-C(3)#1	151.19(10)
C(4)#1-Th(1)-C(3)#1	28.74(9)
C(4)-Th(1)-C(3)#1	88.88(10)
C(5)#1-Th(1)-C(3)#1	47.51(9)
C(5)-Th(1)-C(3)#1	84.13(10)
C(3)-Th(1)-C(3)#1	116.67(14)
C(15)-Th(1)-C(2)	95.05(11)
C(15)#1-Th(1)-C(2)	103.45(11)
C(14)-Th(1)-C(2)	70.02(10)
C(14)#1-Th(1)-C(2)	131.88(10)
C(12)-Th(1)-C(2)	76.96(10)
C(12)#1-Th(1)-C(2)	123.94(10)
C(13)#1-Th(1)-C(2)	141.53(10)
C(13)-Th(1)-C(2)	63.58(10)
C(4)#1-Th(1)-C(2)	108.42(10)
C(4)-Th(1)-C(2)	46.49(9)
C(5)#1-Th(1)-C(2)	111.37(10)
C(5)-Th(1)-C(2)	46.57(9)
C(3)-Th(1)-C(2)	28.64(9)
C(3)#1-Th(1)-C(2)	130.44(10)
C(15)-Th(1)-C(2)#1	103.45(11)
C(15)#1-Th(1)-C(2)#1	95.05(11)
C(14)-Th(1)-C(2)#1	131.88(10)
C(14)#1-Th(1)-C(2)#1	70.02(10)
C(12)-Th(1)-C(2)#1	123.94(10)
C(12)#1-Th(1)-C(2)#1	76.96(10)
C(13)#1-Th(1)-C(2)#1	63.58(10)

C(13)-Th(1)-C(2)#1	141.53(10)
C(4)#1-Th(1)-C(2)#1	46.49(9)
C(4)-Th(1)-C(2)#1	108.42(10)
C(5)#1-Th(1)-C(2)#1	46.57(10)
C(5)-Th(1)-C(2)#1	111.37(10)
C(3)-Th(1)-C(2)#1	130.44(10)
C(3)#1-Th(1)-C(2)#1	28.64(9)
C(2)-Th(1)-C(2)#1	154.80(13)
C(15)-Th(1)-C(1)#1	90.50(11)
C(15)#1-Th(1)-C(1)#1	119.57(11)
C(14)-Th(1)-C(1)#1	119.57(11)
C(14)#1-Th(1)-C(1)#1	90.96(11)
C(12)-Th(1)-C(1)#1	145.10(10)
C(12)#1-Th(1)-C(1)#1	72.59(10)
C(13)#1-Th(1)-C(1)#1	73.48(10)
C(13)-Th(1)-C(1)#1	144.44(10)
C(4)#1-Th(1)-C(1)#1	47.06(9)
C(4)-Th(1)-C(1)#1	95.62(10)
C(5)#1-Th(1)-C(1)#1	28.57(10)
C(5)-Th(1)-C(1)#1	111.85(10)
C(3)-Th(1)-C(1)#1	108.33(10)
C(3)#1-Th(1)-C(1)#1	47.69(9)
C(2)-Th(1)-C(1)#1	136.94(9)
C(2)#1-Th(1)-C(1)#1	28.40(9)
C(15)-Th(1)-C(1)	119.57(11)
C(15)#1-Th(1)-C(1)	90.50(11)
C(14)-Th(1)-C(1)	90.96(11)
C(14)#1-Th(1)-C(1)	119.57(11)
C(12)-Th(1)-C(1)	72.59(10)
C(12)#1-Th(1)-C(1)	145.10(10)
C(13)#1-Th(1)-C(1)	144.44(10)
C(13)-Th(1)-C(1)	73.48(10)
C(4)#1-Th(1)-C(1)	95.61(10)
C(4)-Th(1)-C(1)	47.06(9)
C(5)#1-Th(1)-C(1)	111.85(10)
C(5)-Th(1)-C(1)	28.57(10)

C(3)-Th(1)-C(1)	47.69(9)
C(3)#1-Th(1)-C(1)	108.33(9)
C(2)-Th(1)-C(1)	28.40(9)
C(2)#1-Th(1)-C(1)	136.94(9)
C(1)#1-Th(1)-C(1)	140.05(14)
C(6)-Si(1)-C(7)	109.17(18)
C(6)-Si(1)-C(1)	106.20(17)
C(7)-Si(1)-C(1)	109.51(16)
C(6)-Si(1)-C(8)	106.27(18)
C(7)-Si(1)-C(8)	109.60(19)
C(1)-Si(1)-C(8)	115.84(16)
C(10)-Si(2)-C(9)	109.6(3)
C(10)-Si(2)-C(11)	107.0(2)
C(9)-Si(2)-C(11)	111.0(2)
C(10)-Si(2)-C(3)	112.36(18)
C(9)-Si(2)-C(3)	109.05(18)
C(11)-Si(2)-C(3)	107.88(16)
C(5)-C(1)-C(2)	105.7(3)
C(5)-C(1)-Si(1)	126.8(3)
C(2)-C(1)-Si(1)	124.2(3)
C(5)-C(1)-Th(1)	73.32(18)
C(2)-C(1)-Th(1)	75.01(18)
Si(1)-C(1)-Th(1)	132.78(16)
C(1)-C(2)-C(3)	110.3(3)
C(1)-C(2)-Th(1)	76.59(19)
C(3)-C(2)-Th(1)	74.82(19)
C(4)-C(3)-C(2)	105.6(3)
C(4)-C(3)-Si(2)	126.5(3)
C(2)-C(3)-Si(2)	126.8(3)
C(4)-C(3)-Th(1)	74.84(19)
C(2)-C(3)-Th(1)	76.54(19)
Si(2)-C(3)-Th(1)	123.12(15)
C(5)-C(4)-C(3)	109.4(3)
C(5)-C(4)-Th(1)	75.80(19)
C(3)-C(4)-Th(1)	76.43(19)
C(4)-C(5)-C(1)	108.9(3)

C(4)-C(5)-Th(1)	75.68(19)
C(1)-C(5)-Th(1)	78.10(19)
C(13)-C(12)-C(15)#1	135.3(4)
C(13)-C(12)-Th(1)	76.7(2)
C(15)#1-C(12)-Th(1)	72.3(2)
C(14)-C(13)-C(12)	136.6(4)
C(14)-C(13)-Th(1)	72.7(2)
C(12)-C(13)-Th(1)	74.6(2)
C(13)-C(14)-C(15)	134.0(4)
C(13)-C(14)-Th(1)	78.5(2)
C(15)-C(14)-Th(1)	73.9(2)
C(12)#1-C(15)-C(14)	132.8(4)
C(12)#1-C(15)-Th(1)	78.5(2)
C(14)-C(15)-Th(1)	76.4(2)

Symmetry transformations used to generate equivalent atoms:

#1 -x,y,-z+1/2

Definitions:

$wR2 = [\Sigma[w(F_o^2-F_c^2)^2] / \Sigma[w(F_o^2)^2]]^{1/2}$

$R1 = \Sigma||F_o|-|F_c|| / \Sigma|F_o|$

$Goof = S = [\Sigma[w(F_o^2-F_c^2)^2] / (n-p)]^{1/2}$ where n is the number of reflections and p is the total number of parameters refined.

References

(1) D. L. Clark, T. M. Frankcom, M. M. Miller and J. G. Watkin, *Inorg. Chem.* 1992, **31**, 1628.

(2) D. E. Bergbreiter and J. M. Killough, *J. Am. Chem. Soc.* 1978, **100**, 2126.

(3) J. K. Peterson, M. R. MacDonald, J. W. Ziller and W. J. Evans, *Organometallics* 2013, **32**, 2625.

(4) P. C. Blake, N. M. Edelstein, P. B. Hitchcock, W. K. Kot, M. F. Lappert, G. V. Shalimoff and S. Tian, *J. Organomet. Chem.* 2001, **636**, 124.

(5) V. N. Staroverov, G. E. Scuseria, J. Tao and J. P. Perdew, *J. Chem. Phys.* 2003, **119**, 12129.

(6) A. Schäfer, H. Horn and R. Ahlrichs, *J. Chem. Phys.* 1992, **97**, 2571.
(7) W. Küchle, M. Dolg, H. Stoll and H. Preuss, *J. Chem. Phys*. 1994, **100**, 7535.
(8) X. Cao, M. Dolg, *J. Mol. Struct*. (Theochem) 2004,**673**, 203.
(9) P. Deglmann, K. May, F. Furche and R. Ahlrichs, *Chem. Phys. Lett.* 2004, **384**, 103.
(10) A. Klamt and G. Schueuermann, *J. Chem. Soc., Perkin Trans. 2* 1993, 799.
(11) CRC Handbook of Chemistry and Physics, 81st ed. Lide, D. R., Ed.; CRC Press: Boca Raton, FL, 2008; Chapter 8, p 136.
(12) F. Weigend and R. Ahlrichs, *Phys. Chem. Chem. Phys.* 2005, **7,** 3297.
(13) TURBOMOLE, V6-5; TURBOMOLE GmbH: Karlsruhe, 2013. http://www.turbomole.com.
(14) H. Eshuis, J. Yarkony, F. Furche, *J. Chem. Phys.* 2010, **132**, 234114.
(15) R. Bauernschmitt and R. Ahlrichs, *Chem. Phys. Lett.* 1996, **256**, 454.
(16) J. E. Bates and F. Furche, *J. Chem. Phys.* 2012, **137**, 164105/1.
(17) D. Rappoport and F. Furche, *J. Chem. Phys.* 2010, **133**, 134105/1.
(18) A. Brown, C. M. Kemp, and S. F. Mason, *J. Chem. Soc. A* 1971, 751.
(19) R. S. Mulliken, *J. Chem. Phys.* 1955, **23**, 1833.
(20) APEX2 Version 2014.1-1, Bruker AXS, Inc.; Madison, WI 2014.
(21) SAINT Version 8.34a, Bruker AXS, Inc.; Madison, WI 2013.
(22) Sheldrick, G. M. SADABS, Version 2012/1, Bruker AXS, Inc.; Madison, WI 2012.
(23) Sheldrick, G. M. SHELXTL, Version 2014/2, Bruker AXS, Inc.; Madison, WI 2014.
(24) P. C. Blake, M. F. Lappert, J. L. Atwood and H. Zang, Chem. Commun. 1986, 1148.
(25) International Tables for X-Ray Crystallography 1992, Vol. C., Dordrecht: Kluwer Academic Publishers.
(26) APEX2 Version 2013.10-0, Bruker AXS, Inc.; Madison, WI 2013.
